# Long-Term Exposure to Air Pollution and Risk and Prognosis of Motor Neuron Disease

**DOI:** 10.1001/jamaneurol.2025.5379

**Published:** 2026-01-20

**Authors:** Jing Wu, Andrei Pyko, Charilaos Chourpiliadis, Yihan Hu, Can Hou, Susanna Brauner, Fredrik Piehl, Petter Ljungman, Caroline Ingre, Fang Fang

**Affiliations:** 1 Institute of Environmental Medicine (IMM), Karolinska Institutet, Stockholm, Sweden; 2 Center for Occupational and Environmental Medicine, Stockholm Region, Stockholm, Sweden; 3 Mental Health Center and West China Biomedical Big Data Center, West China Hospital, Sichuan University, Chengdu, China; 4 Med-X Center for Informatics, Sichuan University, Chengdu, China; 5 Department of Clinical Neuroscience, Karolinska Institutet, Stockholm, Sweden; 6 Neuroimmunology Unit, Center for Molecular Medicine, Karolinska Institutet, Stockholm, Sweden; 7 Department of Neurology, Karolinska University Hospital, Stockholm, Sweden; 8 Department of Cardiology, Danderyd Hospital, Stockholm, Sweden

## Abstract

**Importance:**

Air pollution exposure has been associated with an increased risk of neurodegenerative diseases; however, evidence is limited for motor neuron disease (MND), especially regarding disease progression.

**Objective:**

To determine whether long-term exposure to air pollution is associated with the risk and prognosis of MND.

**Design, Setting, and Participants:**

This population-based, nested case-control study used Swedish health register data of incident MND cases diagnosed between 2015 and 2023 with up to 8 years of follow-up. Participants included patients with MND, 5 age- and sex-matched population controls without MND per patient with MND, and full siblings of the patients with MND. Data were analyzed between November 6, 2024, and November 4, 2025.

**Exposures:**

Mean yearly concentrations of particulate matters of 2.5 µm or less, 10 µm or less, or 2.5 to 10 µm in diameter (PM_2.5_, PM_10_, PM_2.5-10_) and nitrogen dioxide (NO_2_) were assessed at the residential address using a spatiotemporal model to approximate accumulated air pollution exposure.

**Main Outcome and Measures:**

Association between air pollution and risk of MND was assessed by comparing cases to both population and sibling controls. Flexible parametric survival models estimated the association between air pollution exposure and the risk of mortality (or use of invasive ventilation) after MND diagnosis (case-only analyses). Based on the rate of decline in the ALS Functional Rating Scale-Revised (ALSFRS-R) score and its subscores after diagnosis, patients were classified into fast (upper 25th percentile) or slow (lower 75th percentile) progression. Logistic regression was used to assess air pollution exposure and the risk of fast progression.

**Results:**

The study included 1463 patients with MND, 7310 population controls, and 1768 sibling controls. The mean (SD) age for all patients with MND was 67.3 (11.7) years, and 814 (55.6) were male. In the population comparison, long-term air pollution was associated with an increased risk of MND; per IQR increase in the 10-year average level, the odds ratio was 1.21 (95% CI, 1.09-1.34) for PM_2.5_, 1.30 (95% CI, 1.19-1.42) for PM_2.5-10_, 1.29 (95% CI, 1.18-1.42) for PM_10_, and 1.20 (95% CI, 1.12-1.29) for NO_2_. A higher level of PM_10_ or NO_2_ was associated with a higher hazard of mortality, whereas a higher level of all PMs was associated with faster functional decline, particularly motor and respiratory functions, after MND diagnosis.

**Conclusions and Relevance:**

The findings of this case-control study suggest that air pollution, even at relatively low levels typical of Sweden, may contribute both to the risk of developing MND and disease prognosis after MND diagnosis.

## Key Points

**Question:**

Is long-term exposure to air pollution associated with the risk and prognosis of motor neuron disease (MND)?

**Findings:**

In this nested case-control study including 1463 patients with newly diagnosed MND, 7310 age- and sex-matched population controls, and 1768 sibling controls, long-term exposure to air pollution was associated with an increased risk of MND as well as increased mortality and faster disease progression after MND diagnosis.

**Meaning:**

These findings suggest that long-term exposure to air pollution may contribute to the development of MND and accelerate disease progression.

## Teaser

This case-control study examines whether long-term exposure to air pollution is associated with the risk and prognosis of motor neuron disease.

## Introduction

Motor neuron disease (MND) refers to a group of progressive neurodegenerative disorders, among which amyotrophic lateral sclerosis (ALS) accounts for 85% to 90% of cases.^1^ ALS is characterized by selective degeneration of both upper and lower motor neurons, resulting in progressive muscle weakness, paralysis, and ultimately respiratory failure. The global incidence of ALS is estimated at 1.5 to 4 cases per 100 000 person-years, with a prevalence of 4 to 6 per 100 000 individuals.^1,2^ The clinical course is highly heterogeneous. While median survival ranges from 20 to 48 months after diagnosis, 10% to 20% of patients survive beyond 10 years.^3,4^ Although the origin of disease for most patients with ALS remains unknown, various environmental exposures, including long-term exposure to air pollution, have been proposed as potential risk factors.^5^

Air pollution is commonly described as a mixture of gaseous pollutants such as nitrogen dioxide (NO_2_) and particulate matter (PM). PM is classified based on its aerodynamic diameter as less than or equal to 2.5 µm (PM_2.5_), less than or equal to 10 µm (PM_10_), or 2.5 to 10 µm (PM_2.5-10_). Accumulating evidence has linked air pollution to a range of adverse health outcomes,^6,7,8^ including neurotoxic effects through neuroinflammation,^9,10,11,12^ oxidative stress,^13^ and blood-brain barrier disruption.^13,14^ Specifically, PMs and other gaseous pollutants could, through translocating across the alveolar-capillary barriers, enter the circulation or, through the olfactory nerve, directly activate glial cells.^15,16^ Once activated, microglia and astrocytes may undergo morphological changes, release chemotactic factors, and generate reactive oxygen species and proinflammatory cytokines.^15^ Moreover, PM_2.5_ may be transported to the terminal bronchioles and alveoli and induce interleukin-6, tumor necrosis factor α, adipokines, and monocyte chemoattractant protein-1.^17^ All of these pathological processes have been proposed to be critically involved in neurodegenerative diseases, including MND.^18^

While some studies find evidence in support of an association between air pollution and MND, findings to date remain mixed.^5^ The conflicting results of the existing literature might be partly due to heterogeneity in sample size, case-control composition, pollutant metrics, and exposure windows.^5^ Moreover, few studies to date have examined whether air pollution influences disease progression among patients with ALS or other MNDs. This study therefore aimed to (1) assess the association between long-term exposure to air pollution and MND risk using a nationwide nested case-control study with both population- and sibling-based comparisons, and (2) evaluate whether air pollution is associated with postdiagnosis disease progression, including patient survival and rate of functional decline.

## Methods

### Study Population

We performed a nested case-control study using data from the Swedish MND Quality Registry^19^ and other Swedish national population and health registers (eFigure 1 in Supplement 1). The MND Quality Registry contains information on approximately 80% of patients with MND in Sweden, including clinical characteristics, biological measurements, and quality of life.^19^ We included 1463 patients with a newly diagnosed MND, including definite, probable, or possible ALS (n = 1057), progressive spinal muscular atrophy (PSMA; n = 61), primary lateral sclerosis (PLS; n = 40), and unspecified MND (n = 305), from January 1, 2015, to July 1, 2023. For each patient, 5 age- and sex-matched MND-free population controls (n = 7310) were randomly selected from the Swedish Total Population Register, using the method of incidence density sampling.^20^ In addition, via Statistics Sweden, we identified 1768 full siblings among 947 patients with MND to serve as sibling controls. The sibling-based comparison was used to alleviate concern of familial confounding due to genetic or nongenetic factors shared between full siblings. We included all eligible siblings as sibling controls for an MND case, if there was more than 1. The diagnosis date of each case was used as the index date for that case and its matched population and sibling controls. A follow-up study of the patients with MND was additionally performed to examine the association of prediagnostic air pollution exposure with patient survival and decline in functional status (ie, case-only analyses). Patients with MND were followed up from the date of diagnosis to death (or the use of invasive ventilation), emigration from Sweden, or end of follow-up (September 1, 2023), whichever occurred first.

The study was approved by the Swedish Ethical Review Authority. Informed consent from study participants was waived by this approval because information was collected from participants on an opt-out basis. This study the Strengthening the Reporting of Observational Studies in Epidemiology (STROBE) reporting guideline.

### Assessment of Air Pollution

We obtained complete residential histories for 1990 to 2022, considering the moving status between years but not within a calendar year, for all study participants from Statistics Sweden. Annual concentrations of PM_2.5_, PM_10_, PM_2.5-10_, and NO_2_ were assessed for 2005 to 2019 using validated satellite-based spatiotemporal models.^21^ These models use machine learning, multistage procedures that integrate dispersion models, satellite observations, land-use and meteorological data, residential location, and road-traffic information, and they have been calibrated against measurements from 180 monitoring stations covering all counties in Sweden.^21^ Predicted concentrations are mapped across Sweden on a 1 × 1-km grid and show moderate to high correlations with monitoring data.^21^ Because model outputs were not available for 2020 to 2022, we used the 2019 predictions for those years because concentrations were relatively stable during 2005 to 2019 (eFigure 2 in Supplement 1). Cumulative exposure to different pollutants was calculated as the average concentrations during the 1-, 3-, 5-, and 10-year periods preceding the year of index date. The correlation matrix of these average pollutant concentrations is shown in eFigure 3 in Supplement 1.

### Assessment of Disease Progression, Invasive Ventilation, and Mortality

The ALS Functional Rating Scale-Revised (ALSFRS-R) comprises 12 items covering 4 functional domains: bulbar, fine motor, gross motor, and respiratory functions. Each item is scored 0 to 4, giving a total score of 0 to 48 and 4 domain-specific scores of 0 to 12; higher scores indicate better preserved function. Patients were assessed at diagnosis and follow-up visits (every 6 months). The information on use of invasive ventilation was ascertained from the Swedish MND Quality Registry. Date of death was obtained from the Causes of Death Register.

### Covariates

Data on date of birth and sex were obtained from the Total Population Register. Information on occupation, education, household disposable income, and country of birth was collected from the Swedish Censuses (1965-1990) and the Longitudinal Integrated Database for Health Insurance and Labour Market Studies (1990-2023). Mean neighborhood income during 2015 to 2023 was obtained from Statistics Sweden based on Demographic Statistical Areas (DeSO), which divide Sweden into approximately 6000 areas with a population size of 700 to 2700 residents. A 5-year average neighborhood income before the index date was calculated for each participant to capture socioeconomic disparities at the neighborhood level. Finally, we calculated urbanicity level for the residential area where the study participants lived longest during the 10 years before the index date based on the DeSO categories, including outside major population concentrations or urban areas; in a population concentration or urban area, but not in the municipality’s central area; and in the municipality’s central area.

Clinical characteristics of the patients with MND were identified from the MND Quality Registry. Diagnostic delay was defined as the time interval between symptom onset and date of diagnosis. The onset site was classified as bulbar, spinal, or other. Disease progression rate at diagnosis was calculated by subtracting the ALSFRS-R score at diagnosis from the maximum score of 48 and dividing the result by the diagnostic delay in months. Additional variables, including family history, body mass index (BMI; calculated as weight in kilograms divided by height in meters squared) at diagnosis, and gastrostomy placement, were also retrieved.

### Statistical Analysis

Analyses were conducted between November 6, 2024, and November 4, 2025. Characteristics of the patients with MND and controls at the index date were described and compared using χ^2^ tests for categorical variables and *t* tests for continuous variables. Odds ratios (ORs) of MND and its subtypes with 95% CIs in relation to air pollution exposure were calculated by conditional logistic regression in the population comparison after adjustment for age at index date and sex (matching factors), as well as for country of birth, education, occupation, household disposable income, and 5-year average neighborhood income before the index date. The ORs approximate the hazard ratios (HRs) derived from the cohort, giving rise to the nested case-control study due to the use of incidence density sampling. We then combined a marginalized between-within framework with logistic regression for the analyses of the sibling comparison to better control for familial confounding and maximize statistical power, after adjustment for the same covariates and within-family averages of these covariates.^22^ In the population comparison, we conducted 2 sensitivity analyses. First, we excluded MND cases diagnosed between 2021 and 2023 and their controls to assess the soundness of imputing air pollution data during 2020 and 2022 by the 2019 predictions. Second, we additionally adjusted for the urbanicity level of the longest-living area to assess potential confounding by urbanicity.

To assess long-term exposure to air pollution in relation to mortality (or use of invasive ventilation) after MND diagnosis, we estimated the overall HRs using flexible parametric survival models with 2 degrees of freedom for time, which do not require the proportional hazard function assumption as Cox models.^23^ Analyses were adjusted for age at diagnosis and sex in model 1 and additionally for country of birth, education, occupation, household disposable income, 5-year average neighborhood income, diagnostic delay, and ALSFRS-R score at diagnosis in model 2.

To explore the association of air pollution with functional decline after diagnosis, we used logistic regression to assess the OR of fast progression in the overall or domain-specific ALSFRS-R scores. Rates of decline in total and domain-specific scores over time were estimated using linear mixed-effects models with random intercepts and slopes to derive individual slopes; patients in the top quartile of decline were classified as having fast progression, and those in the lower quartiles as having slow progression (eFigure 4 in Supplement 1). To address potential nonlinear decline in ALSFRS-R scores over time, we performed a sensitivity analysis by including fixed and random quadratic time terms in the mixed models.

Concentrations of air pollutants were modeled per 1-IQR increment: 2 µg/m^3^ for PM_2.5_, 2.8 µg/m^3^ for PM_2.5-10_, 4 µg/m^3^ for PM_10_, and 8 µg/m^3^ for NO_2_. In the main analysis, we included all patients with MND; in the secondary analyses, we focused on patients with ALS (including PSMA) to examine whether the results pattern would differ between ALS and other MNDs. Sensitivity analyses were conducted separating mortality and invasive ventilation as 2 independent outcomes and without adjusting for diagnostic delay and ALSFRS-R score at diagnosis. A 2-sided *P* < .05 indicated statistical significance. All analyses were performed in Stata MP, version 18 (StataCorp LLC).

## Results

### Characteristics of the Study Participants

A total of 1463 patients with newly diagnosed MND, 7310 population controls, and 1768 sibling controls were included in the study. Among the 1463 patients with MND in the population comparison, the mean (SD) age at the index date was 67.3 (11.7) years; 649 (44.4%) were female and 814 (55.6%) were male (Table 1). The average concentrations of all 4 air pollutants at different exposure windows were higher among the cases than controls. Similar, although slightly diminished, differences were observed in the sibling comparison.

**Table 1.** Characteristics of the Study Participants at the Index Date

Characteristics	Population comparison			Sibling comparison		
	MND cases (n = 1463)	Population controls (n = 7310)	*P* value	MND cases (n = 947)	Sibling controls (n = 1768)	*P* value
Age at index date, mean (SD)	67.3 (11.7)	67.3 (11.6)	>.99	66.1 (11.4)	65.5 (12.3)	.24
Sex, No. (%)						
Male	814 (55.6)	4066 (55.6)	.99	536 (56.6)	854 (48.3)	<.001
Female	649 (44.4)	3244 (44.4)		411 (43.4)	914 (51.7)	
Household disposable income, No. (%)						
Lowest 25%	328 (22.4)	2040 (27.9)	<.001	185 (19.5)	406 (23.0)	<.001
25%-50%	333 (22.8)	1637 (22.4)		190 (20.1)	375 (21.2)	
50%-75%	320 (21.9)	1580 (21.6)		220 (23.2)	380 (21.5)	
Highest 75%	482 (32.9)	2053 (28.1)		352 (37.2)	586 (33.1)	
Unknown	NA	NA		NA	21 (1.2)	
Occupation, No. (%)						
Occupation without educational requirement	62 (4.2)	443 (6.1)	<.001	34 (3.6)	90 (5.1)	<.001
Occupation requiring high school degree	634 (43.3)	3504 (47.9)		415 (43.8)	796 (45.0)	
Occupation requiring university studies ≤3 y	265 (18.1)	1069 (14.6)		188 (19.9)	284 (16.1)	
Occupation requiring university studies >3 y	361 (24.7)	1444 (19.8)		253 (26.7)	421 (23.8)	
Missing	141 (9.6)	850 (11.6)		57 (6.0)	177 (10.0)	
Education, No. (%)						
<9 y	152 (10.4)	979 (13.4)	<.001	73 (7.7)	174 (9.8)	.08
9-10 y	147 (10.0)	792 (10.8)		97 (10.2)	186 (10.5)	
Upper secondary education	609 (41.6)	3180 (43.5)		400 (42.2)	781 (44.2)	
Postsecondary <2 y	82 (5.6)	356 (4.9)		64 (6.8)	98 (5.5)	
Postsecondary ≥2 y	439 (30.0)	1849 (25.3)		297 (31.4)	488 (27.6)	
Postgraduate education	26 (1.8)	80 (1.1)		14 (1.5)	28 (1.6)	
Missing	8 (0.5)	74 (1.0)		2 (0.2)	13 (0.7)	
Country of birth, No. (%)						
Sweden	1272 (86.9)	6220 (85.1)	.07	916 (96.7)	1713 (96.9)	.44
Other Nordic countries	60 (4.1)	284 (3.9)		17 (1.8)	22 (1.2)	
Other European countries	72 (4.9)	391 (5.3)		12 (1.3)	24 (1.4)	
Non-European countries	59 (4.0)	415 (5.7)		2 (0.2)	9 (0.5)	
Mean neighborhood income, thousand SEK^a^	340.6 (297.6-402.5)	319.8 (283.5-366.1)	<.001	342.9 (303.1-405.7)	328.2 (292.6-378.7)	<.001
Urbanicity of the longest living area, No. (%)						
Rural area	231 (15.8)	1466 (20.1)	<.001	175 (18.5)	365 (20.6)	.37
Urban area	112 (7.7)	673 (9.2)		84 (8.9)	161 (9.1)	
Central area	1120 (76.6)	5170 (70.7)		688 (72.7)	1242 (70.2)	
Air pollutants, mean (SD), µg/m^3^						
1-y Average PM_2.5_	6.8 (1.6)	6.7 (1.7)	.07	6.7 (1.6)	6.6 (1.6)	.02
1-y Average PM_10_	14.4 (3.0)	13.9 (3.1)	<.001	14.2 (3.0)	13.8 (3.0)	.005
1-y Average PM_2.5-10_	7.3 (2.2)	6.9 (2.1)	<.001	7.1 (2.2)	6.9 (2.1)	.02
1-y Average NO_2_	13.6 (7.4)	12.3 (6.8)	<.001	13.2 (7.4)	12.3 (7.2)	.002
3-y Average PM_2.5_	7.0 (1.5)	6.9 (1.6)	.04	7.0 (1.5)	6.9 (1.5)	.13
3-y Average PM_10_	14.6 (2.9)	14.2 (2.9)	<.001	14.4 (2.9)	14.2 (2.8)	.02
3-y Average PM_2.5-10_	7.5 (2.2)	7.1 (2.0)	<.001	7.3 (2.1)	7.1 (2.0)	.05
3-y Average NO_2_	13.6 (7.3)	12.3 (6.8)	<.001	13.1 (7.3)	12.4 (7.1)	.009
5-y Average PM_2.5_	7.3 (1.4)	7.2 (1.5)	.02	7.3 (1.4)	7.2 (1.5)	.30
5-y Average PM_10_	15.0 (2.8)	14.5 (2.9)	<.001	14.7 (2.8)	14.5 (2.8)	.048
5-y Average PM_2.5-10_	7.6 (2.1)	7.2 (1.9)	<.001	7.4 (2.0)	7.3 (2.0)	.06
5-y Average NO_2_	13.6 (7.3)	12.3 (6.7)	<.001	13.1 (7.3)	12.4 (7.1)	.02
10-y Average PM_2.5_	8.1 (1.3)	8.0 (1.4)	<.001	8.1 (1.3)	8.0 (1.4)	.23
10-y Average PM_10_	15.8 (2.9)	15.2 (2.9)	<.001	15.5 (2.9)	15.3 (2.9)	.04
10-y Average PM_2.5-10_	7.8 (2.2)	7.4 (1.9)	<.001	7.7 (2.1)	7.5 (2.1)	.047
10-y Average NO_2_	13.7 (7.2)	12.4 (6.5)	<.001	13.2 (7.3)	12.5 (7.0)	.02
BMI at diagnosis, mean (SD)	23.9 (4.2)	NA	NA	23.8 (4.0)	NA	NA
ALSFRS-R at diagnosis, mean (SD)	36.4 (8.2)	NA	NA	37.1 (8.1	NA	NA
Diagnostic delay, mean (SD) mo	17.0 (19.6)	NA	NA	17.6 (20.3	NA	NA
Progression rate at diagnosis, median (IQR), points per month	0.7 (0.4-1.4)	NA	NA	0.7 (0.3-1.3)	NA	NA
Family history, No. (%)	60 (10.8)	NA	NA	40 (11.7)	NA	NA
Onset site, No. (%)						
Bulbar	276 (34.2)	NA	NA	163 (33.1)	NA	NA
Spinal	468 (58.0)	NA	NA	289 (58.6)	NA	NA
Other	63 (7.8)	NA	NA	41 (8.3)	NA	NA
Invasive ventilation	20 (1.4)	NA	NA	15 (1.6)	NA	NA
Gastrostomy, No. (%)						
PEG	287 (19.6)	NA	NA	187 (19.7)	NA	NA
RIG	10 (0.7)	NA	NA	6 (0.6)	NA	NA
Wizelfistel	1 (0.1)	NA	NA	0	NA	NA
None	1165 (79.6)	NA	NA	754 (79.6)	NA	NA

Abbreviations: ALSFRS-R, Amyotrophic Lateral Sclerosis Functional Rating scale-revised; BMI, body mass index (calculated as weight in kilograms divided by height in meters squared); MND, motor neuron disease; NA, not applicable; NO_2_, nitrogen dioxide; PEG, percutaneous endoscopic gastrostomy; PM, particulate matter; RIG, radiologically inserted gastrostomy; SEK, Swedish krona.

^a^ Mean neighborhood income was calculated as the 5-year average before the year of the index date.

### Air Pollution and the Risk of MND Diagnosis

A positive association between the 1-, 3-, 5-, and 10-year average concentrations of all air pollutants and an increased risk of MND was evident in the population comparison (Figure). Notably, the risk of MND increased with PM_2.5,_ PM_2.5-10_, PM_10,_ and NO_2_ in both the short- and long-time frames. Per IQR increment in the 1-year average exposure, the ORs were 1.12 (95% CI, 1.02-1.23) for PM_2.5_, 1.25 (95% CI, 1.15-1.36) for PM_2.5-10_, 1.25 (95% CI, 1.14-1.36) for PM_10_, and 1.20 (95% CI, 1.12-1.29) for NO_2_. The corresponding ORs for the 10-year average exposure were 1.21 (95% CI, 1.09-1.34) for PM_2.5_, 1.30 (95% CI, 1.19-1.42) for PM_2.5-10_, 1.29 (95% CI, 1.18-1.42) for PM_10_, and 1.20 (95% CI, 1.12-1.29) for NO_2_. Similar results were noted for ALS but not PLS or other MND (eTable 1 in Supplement 1). The results were overall robust after excluding MND cases diagnosed in 2021 to 2023 and their controls from the analysis or additional adjustment for urbanicity level of the longest-living area (eTable 2 in Supplement 1).

**Figure.**
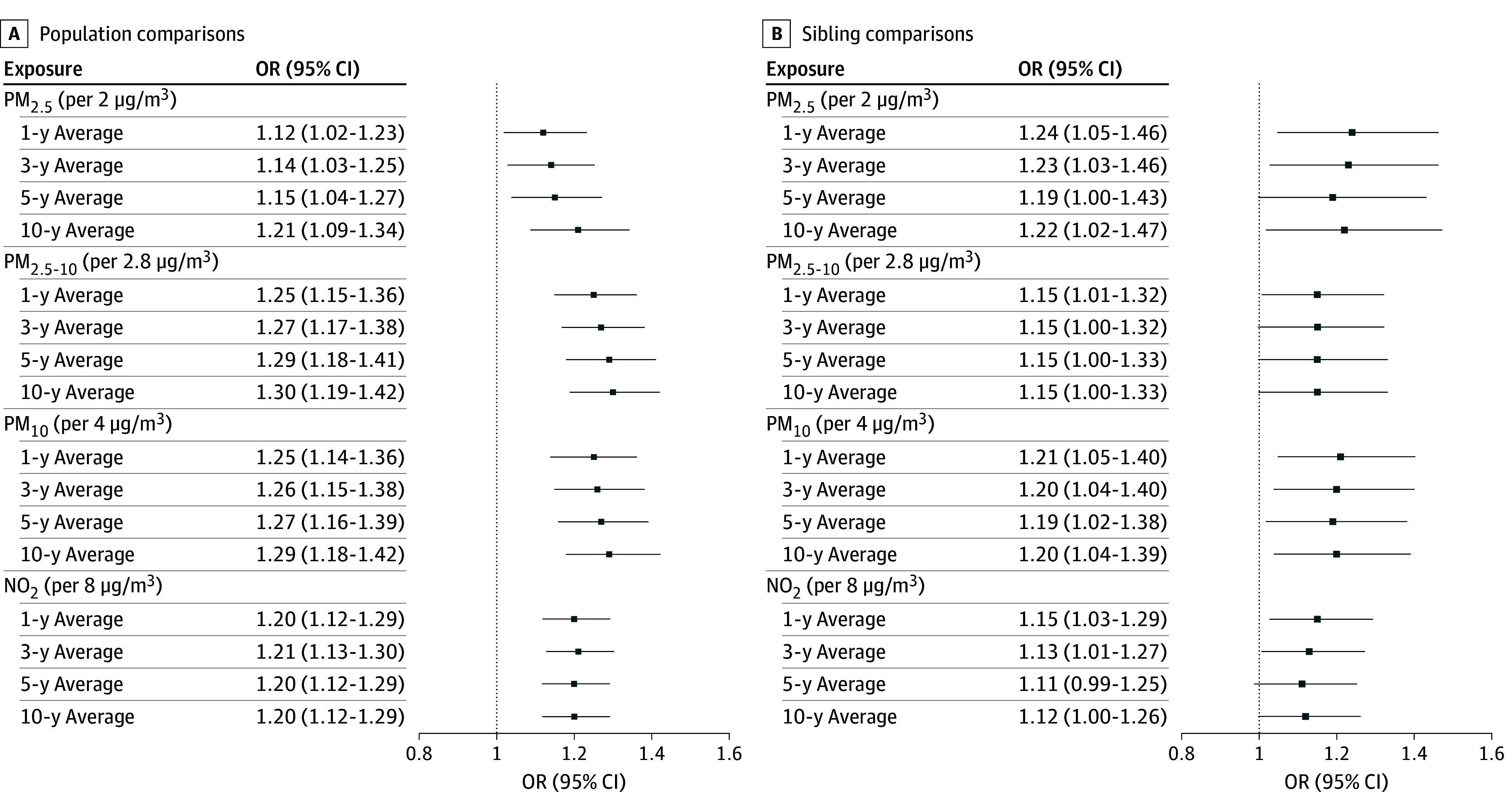
Long-Term Exposure to Air Pollution and the Risk of Motor Neuron Disease In the population comparison, all analyses were adjusted for age at index date and sex (matching factors), as well as country of birth, education, occupation, household disposable income, and 5-year average neighborhood income before the index date. In the sibling comparison, analyses were adjusted for the same covariates and for within-family averages. OR indicates odds ratio; PM, particulate matter; NO_2_, nitrogen dioxide.

Such associations were also observed in the sibling comparison. Per IQR increment in the 10-year average exposure, the ORs were 1.22 (95% CI, 1.02-1.47) for PM_2.5_, 1.15 (95% CI, 1.00-1.33) for PM_2.5-10_, 1.20 (95% CI, 1.04-1.39) for PM_10_, and 1.12 (95% CI, 1.00-1.26) for NO_2_.

### Air Pollution and Survival in Patients With MND

We observed an elevated hazard of mortality (or use of invasive ventilation) among patients with MND in association with a higher level of PM_10_ or NO_2_ in all exposure windows (Table 2). The greatest increment in hazard was noted in association with PM_10_ and NO_2_ during the year before diagnosis (HR, 1.30 [95% CI, 1.12-1.50]; and 1.23 [95% CI, 1.10-1.36] per IQR increment, respectively). When separately analyzing mortality and invasive ventilation as different outcomes, the HRs were higher for invasive ventilation than mortality (eTable 3 in Supplement 1). Analyses not adjusted for diagnostic delay and ALSFRS-R score at diagnosis showed generally similar results (eTable 4 in Supplement 1).

**Table 2.** Association of Long-Term Exposure to Air Pollution With Mortality (or Use of Invasive Ventilation) After Motor Neuron Disease Diagnosis

Air pollutants and exposure windows	HR (95% CI)^a^		
	Crude model	Model 1	Model 2
**PM_2.5_ (per IQR: 2 µg/m^3^)**			
1-y Average	0.95 (0.86-1.04)	0.97 (0.89-1.06)	1.04 (0.91-1.19)
3-y Average	0.89 (0.81-0.99)	0.92 (0.83-1.01)	1.01 (0.87-1.17)
5-y Average	0.90 (0.81-1.00)	0.93 (0.84-1.03)	1.02 (0.88-1.19)
10-y Average	0.95 (0.85-1.07)	0.97 (0.87-1.09)	1.02 (0.85-1.21)
**PM_2.5-10_ (per IQR: 2.8 µg/m^3^)**			
1-y Average	1.12 (1.02-1.22)	1.14 (1.05-1.25)	1.13 (1.00-1.27)
3-y Average	1.13 (1.03-1.24)	1.16 (1.06-1.27)	1.12 (0.98-1.27)
5-y Average	1.13 (1.03-1.24)	1.17 (1.07-1.28)	1.10 (0.97-1.26)
10-y Average	1.14 (1.05-1.25)	1.18 (1.08-1.29)	1.11 (0.97-1.26)
**PM_10_ (per IQR: 4 µg/m^3^)**			
1-y Average	1.11 (1.00-1.22)	1.13 (1.03-1.24)	1.30 (1.12-1.50)
3-y Average	1.09 (0.99-1.21)	1.12 (1.02-1.24)	1.26 (1.08-1.46)
5-y Average	1.09 (0.98-1.21)	1.12 (1.01-1.24)	1.21 (1.04-1.40)
10-y Average	1.12 (1.01-1.24)	1.15 (1.04-1.27)	1.16 (1.01-1.35)
**NO_2_ (per IQR: 8 µg/m^3^)**			
1-y Average	1.14 (1.06-1.23)	1.14 (1.06-1.23)	1.23 (1.10-1.36)
3-y Average	1.13 (1.05-1.23)	1.14 (1.06-1.23)	1.19 (1.08-1.33)
5-y Average	1.13 (1.05-1.23)	1.15 (1.06-1.24)	1.16 (1.05-1.29)
10-y Average	1.14 (1.05-1.23)	1.16 (1.07-1.25)	1.13 (1.02-1.25)

Abbreviations: ALSFRS-R, Amyotrophic Lateral Sclerosis Functional Rating Scale-Revised; HR, hazard ratio; NO_2_, nitrogen dioxide; PM, particulate matter.

^a^ Model 1 was adjusted for age at diagnosis and sex; model 2 was additionally adjusted for country of birth, education, occupation, household disposable income, 5-year average neighborhood income, diagnostic delay, and ALSFRS-R score at diagnosis.

### Air Pollution and Functional Decline After MND Diagnosis

Characteristics of patients with slow and fast progression are shown in eTable 5 in Supplement 1. A higher level of 10-year average exposure to PMs was associated with a higher odds of fast progression concerning the total ALSFRS-R score (OR per IQR increment in PM_2.5_, 1.34 [95% CI, 1.02-1.77]; PM_2.5-10_, 1.31 [95% CI, 1.06-1.61]; and PM_10_, 1.30 [95% CI, 1.03-1.66]) (Table 3). Positive associations were also noted between higher levels of PM_2.5-10_, PM_10_, and NO_2_ in all exposure windows and having fast progression in fine motor function. Further, the 5- and 10-year average exposure to PM_2.5_ was associated with an increased odds of fast progression in respiratory function (OR, 1.31 [95% CI, 1.01-1.71] and OR, 1.41 [95% CI, 1.04-1.91] per IQR increment, respectively), whereas the 3- and 5-year average exposure to PM_2.5-10_ was associated with an elevated odds of fast progression in gross motor function (OR, 1.30 [95% CI, 1.06-1.60] and OR, 1.27 [95% CI, 1.03-1.57] per IQR increment, respectively). No association was noted for bulbar function.

**Table 3.** Long-Term Exposure to Air Pollution and Risk of Fast Progression in the Overall or Domain-Specific Scores of ALSFRS-R After Motor Neuron Disease Diagnosis

Air pollutants and exposure windows	OR (95% CI)^a^				
	Total score	Bulbar	Motor		Respiratory
			Fine	Gross	
**PM_2.5_ (per IQR: 2 µg/m^3^)**					
1-y Average	0.96 (0.76-1.20)	1.00 (0.80-1.26)	1.06 (0.83-1.35)	0.90 (0.71-1.14)	1.10 (0.86-1.40)
3-y Average	1.14 (0.90-1.44)	0.96 (0.76-1.22)	1.20 (0.93-1.56)	1.01 (0.78-1.29)	1.26 (0.97-1.63)
5-y Average	1.22 (0.95-1.55)	1.01 (0.79-1.29)	1.27 (0.98-1.65)	1.07 (0.83-1.38)	1.31 (1.01-1.71)
10-y Average	1.34 (1.02-1.77)	1.08 (0.82-1.43)	1.34 (1.00-1.81)	1.17 (0.87-1.57)	1.41 (1.04-1.91)
**PM_2.5-10_ (per IQR: 2.8 µg/m^3^)**					
1-y Average	1.16 (0.96-1.41)	1.04 (0.85-1.26)	1.40 (1.14-1.71)	1.20 (0.98-1.46)	1.16 (0.94-1.43)
3-y Average	1.24 (1.01-1.51)	1.11 (0.90-1.36)	1.44 (1.17-1.79)	1.30 (1.06-1.60)	1.17 (0.94-1.46)
5-y Average	1.27 (1.04-1.57)	1.12 (0.91-1.38)	1.43 (1.15-1.77)	1.27 (1.03-1.57)	1.12 (0.90-1.40)
10-y Average	1.31 (1.06-1.61)	1.13 (0.92-1.39)	1.35 (1.09-1.67)	1.23 (1.00-1.52)	1.06 (0.85-1.32)
**PM_10_ (per IQR: 4 µg/m^3^)**					
1-y Average	1.07 (0.85-1.34)	1.06 (0.85-1.33)	1.31 (1.03-1.67)	1.02 (0.80-1.29)	1.04 (0.81-1.33)
3-y Average	1.20 (0.95-1.53)	1.07 (0.84-1.36)	1.39 (1.07-1.80)	1.14 (0.88-1.46)	1.05 (0.81-1.37)
5-y Average	1.23 (0.96-1.56)	1.05 (0.82-1.33)	1.36 (1.05-1.76)	1.12 (0.87-1.43)	1.04 (0.80-1.35)
10-y Average	1.30 (1.03-1.66)	1.10 (0.86-1.39)	1.33 (1.04-1.72)	1.15 (0.90-1.47)	1.05 (0.81-1.35)
**NO_2_ (per IQR: 8 µg/m^3^)**					
1-y Average	1.18 (0.99-1.40)	1.15 (0.97-1.36)	1.36 (1.14-1.64)	1.12 (0.94-1.34)	1.02 (0.84-1.23)
3-y Average	1.16 (0.98-1.38)	1.15 (0.97-1.37)	1.32 (1.10-1.59)	1.15 (0.96-1.38)	0.99 (0.82-1.20)
5-y Average	1.15 (0.97-1.37)	1.13 (0.95-1.34)	1.31 (1.09-1.57)	1.15 (0.96-1.37)	0.99 (0.82-1.20)
10-y Average	1.13 (0.95-1.35)	1.12 (0.94-1.33)	1.25 (1.04-1.50)	1.12 (0.93-1.34)	0.97 (0.80-1.18)

Abbreviations: ALSFRS-R, Amyotrophic Lateral Sclerosis Functional Rating scale-revised; OR, odds ratio; PM, particulate matter; NO_2_, nitrogen dioxide.

^a^ Models were adjusted for age at diagnosis, sex, education, occupation, household disposable income, 5-year average neighborhood income, diagnostic delay, and ALSFRS-R score at diagnosis.

Restricting the analyses to patients with ALS (including PSMA) rendered similar results both in terms of mortality (eTable 6 in Supplement 1) and functional decline (eTable 7 in Supplement 1). Similar patient grouping was observed in the sensitivity analysis of potentially nonlinear declining of ALSFRS-R scores (eFigure 5 in Supplement 1), leading further to similar associations between air pollution and fast progression (eTable 8 in Supplement 1). Similar results were noted in analyses not adjusted for diagnostic delay and ALSFRS-R score at diagnosis (eTable 9 in Supplement 1).

## Discussion

We conducted a nationwide case-control study with access to detailed information on residential exposures to air pollutants to address not only the risk but also disease progression of MND. We found a consistently increased risk of MND in relation to a higher level of PM_2.5_, PM_2.5-10_, PM_10_, and NO_2_ in different exposure windows up to 10 years before diagnosis. Long-term exposure to air pollution was further found to be associated with an increased risk of mortality and need of invasive ventilation after MND diagnosis. A faster loss of motor and respiratory functions, but not bulbar function, was also evident among patients with MND with a higher 10-year average exposure to all PMs.

In contrast to the somewhat conflicting results of existing literature,^24,25,26,27,28,29^ our study showed a consistent association between air pollution exposure and a higher risk of MND across multiple pollutants and exposure windows. Varying study sample size, validity of the case-control selection, and air pollution measures (eg, choice of pollutants, geographic spread, severity of pollution, and duration of exposure) could all contribute to conflicting results. For instance, a large Danish case-control study explored several exposure windows and suggested that exposure to PM_2.5_ within 6 years before diagnosis might be more relevant for MND risk than other exposure windows.^25^ In the population comparison of the present study, we found a consistently increased risk of MND in association with 1-, 3-, 5-, and 10-year exposure to air pollutants. However, the associations appeared to be slightly stronger for the 10-year exposure window, shedding light on the accumulated risk of poor air quality. Importantly, similar positive associations were noted between different pollutants and MND risk in the sibling comparison, although the point estimates diminished slightly for some of the pollutants. Such a diminishment might indicate the presence of confounding due to shared genetic and environmental factors between the siblings or a potential overmatching of the sibling comparison (ie, siblings were also similar regarding exposure to air pollution). Nonetheless, given that the risk genes of MND (ie, *SOD1* and *C9orf72*) converge on similar functional pathways, including oxidative stress and neuroinflammation, as air pollution, there might be potential interactions between such risk genes and air pollution.^30^ As we did not have genotyping data for the controls in the present study, we encourage future studies to examine whether the link between air pollution and MND risk varies between individuals with or without risk genes.

Key strengths of the study are the nationwide design, the large sample size, the detailed data on clinical characteristics at the time of diagnosis and during follow-up for the patients with MND, and the adjustment for important covariables at individual and neighborhood levels to reduce risk of confounding. Unlike studies using air pollution measurement at one time or with low spatial resolutions,^26,27^ we had access to air quality data with a high spatial resolution and over an extended period of time, which allowed separate analyses of 4 key air pollutants and 4 exposure windows. Further, availability of repeated measures of functional status allowed us to explore the impact of air pollution on functional decline overall and in 4 different domains among patients with MND.

This study is also among the first to demonstrate a higher risk of mortality or invasive ventilation use in association with a higher prediagnostic long-term exposure to PM_10_ and NO_2_ among patients with MND. In line with this finding, a US nested case-control study of 256 ALS cases identified from death records and 2486 matched healthy controls also found a 7% increased risk of ALS mortality per 1-µg/m^3^ increase in the 7.5-year average concentration of PM_2.5-10_, but no increase with PM_2.5_ or PM_10_.^31^ In the main analysis, the associations were only statistically significant for PM_10_ and NO_2_, which may indicate stronger effects of local traffic emissions compared with long-range transported PM predominantly affecting the variation in PM_2.5_; however, in the sensitivity analysis of mortality and invasive ventilation as 2 separate outcomes, the difference between the 4 pollutants diminished. Intriguingly, in this latter analysis, higher HRs were observed for air pollutants, especially PM_2.5_, and use of invasive ventilation compared with mortality, although the findings for invasive ventilation were not always statistically significant due to the small number of individuals. These results may be partly attributed to a stronger association between fine inhalable particles and respiratory function impairment than gaseous pollution. Nevertheless, the small number of patients with invasive ventilation in the study does not preclude the possibility of chance finding.

Further, we found that the 10-year prediagnostic average concentrations of different PMs were associated with an accelerated decline of motor and respiratory, but not bulbar, functions after MND diagnosis. This discrepancy may potentially relate to differences in the neuroanatomical basis of the respective functional domains, including differences in sensitivity to the mechanisms triggered by air pollution. Exposure to PMs has indeed been associated with poorer gross motor development in children^32^ as well as reduced lung function and increased susceptibility to respiratory infections and chronic lung diseases in adults.^33,34,35^ Reduced lung function, infections, and chronic lung diseases have all been shown to increase the risk for poorer outcomes in ALS.^36^ In sum, air pollution may convey an increased risk of mortality and faster functional decline after MND diagnosis through direct and indirect pathways.

### Limitations

This study has some limitations. First, we had no information on indoor, workplace, or leisure time exposure to air pollution. Although outdoor and indoor air pollutions are highly correlated with one another due to the penetration of air pollutants,^37^ additional factors are known to influence indoor exposure, such as ventilation status or type of cooking fuel. Some degree of exposure misclassification is therefore unavoidable. Nonetheless, such misclassification is unlikely systematically related to either outdoor exposure or MND diagnosis. Any bias related to such misclassification would therefore most likely attenuate the results toward the null. Second, our study lacks data on source-specific PMs, limiting the possibility of studying whether the findings are specific to PMs from different sources, such as traffic, industrial sources, or wildfires. Third, given the register-based nature of the present study, we had no data on lifestyle factors such as smoking. However, as the rate of smoking in the Swedish population is approximately 5% (ie, the lowest in Europe), lack of adjustment for smoking is unlikely to substantially bias the results. Nonetheless, smoking was not a residual confounder or modifier in the association between air pollution and ALS in a previous study.^26^ Fourth, we did not have access to air pollution data for 2020 to 2022; the number of monitoring stations varied across the country (ie, there were more stations in more populated areas); and the level of air pollution was shown to have reduced during the COVID-19 pandemic.^38^ However, although these limitations may have introduced error in our exposure assessment, they are again most likely nondifferential between cases and controls. Fifth, because the analyses were mostly exploratory in nature, we did not correct for multiple testing; therefore, the possibility of false positive findings cannot be excluded.

## Conclusions

This case-control study supports the notion that air pollution, even at relatively low exposure levels typical of Sweden, contributes both to the risk of developing MND and to disease prognosis after MND diagnosis. These results highlight the public health importance of improving air quality to reduce the risk of neurodegenerative diseases and to improve the outcome of patients with these diseases.

## Notes

Supplement 1. **eFigure 1.** Study design **eFigure 2. **Temporal trend of yearly average concentrations of different air pollutants from 2005 to 2019 **eFigure 3. **The correlation matrix of air pollution at different exposure windows **eFigure 4. **Observed and predicted individual linear trajectories of ALSFR-S overall and domain-specific scores by time since diagnosis **eFigure 5. **Observed and predicted individual nonlinear trajectories of ALSFR-S overall and domain-specific scores by time since diagnosis **eTable 1. **Long-term exposure to air pollution and risk of MND subtypes – analyses of the population comparison **eTable 2. **Long-term exposure to air pollution and risk of MND – sensitivity analyses in the population comparison **eTable 3. **Long-term exposure to air pollution in relation to mortality and use of invasive ventilation after MND diagnosis **eTable 4. **Long-term exposure to air pollution in relation to mortality (or use of invasive ventilation) after MND diagnosis – analysis not adjusted for diagnostic delay and ALSFRS-R score at diagnosis **eTable 5. **Characteristics of slow and fast progressors in the overall or domain-specific scores of ALSFRS-R **eTable 6. **Long-term exposure to air pollution in relation to mortality (or use of invasive ventilation) after MND diagnosis – analysis focusing on ALS (including PSMA) patients **eTable 7. **Long-term exposure to air pollution and risk of being a fast progressor in the overall or domain-specific scores of ALSFRS-R after MND diagnosis – analysis focusing on ALS (including PSMA) patients **eTable 8. **Long-term exposure to air pollution and risk of being a fast progressor in the overall or domain-specific scores of ALSFRS-R after MND diagnosis – analysis using nonlinear model for fast progressor categorization **eTable 9. **Long-term exposure to air pollution and risk of being a fast progressor in the overall or domain-specific scores of ALSFRS-R after MND diagnosis – analysis not adjusted for diagnostic delay and ALSFRS-R score at diagnosis

Supplement 2.
Data Sharing Statement
