# Supplementary material for: Long-Term Exposure to Air Pollution and Risk and Prognosis of Motor Neuron Disease
Source: JAMA Neurol. 2026 Jan 20;83(3):213–22. doi: 10.1001/jamaneurol.2025.5379 (PMC12820776; doi:10.1001/jamaneurol.2025.5379)
Supplement: Supplement 1. — eFigure 1. Study design eFigure 2. Temporal trend of yearly average concentrations of different air pollutants from 2005 to 2019 eFigure 3. The correlation matrix of air pollution at different exposure windows eFigure 4. Observed and predicted individual linear trajectories of ALSFR-S overall and domain-specific scores by time since diagnosis eFigure 5. Observed and predicted individual nonlinear trajectories of ALSFR-S overall and domain-specific scores by time since diagnosis eTable 1. Long-term exposure to air pollution and risk of MND subtypes – analyses of the population comparison eTable 2. Long-term exposure to air pollution and risk of MND – sensitivity analyses in the population comparison eTable 3. Long-term exposure to air pollution in relation to mortality and use of invasive ventilation after MND diagnosis eTable 4. Long-term exposure to air pollution in relation to mortality (or use of invasive ventilation) after MND diagnosis – analysis not adjusted for diagnostic delay and ALSFRS-R score at diagnosis eTable 5. Characteristics of slow and fast progressors in the overall or domain-specific scores of ALSFRS-R eTable 6. Long-term exposure to air pollution in relation to mortality (or use of invasive ventilation) after MND diagnosis – analysis focusing on ALS (including PSMA) patients eTable 7. Long-term exposure to air pollution and risk of being a fast progressor in the overall or domain-specific scores of ALSFRS-R after MND diagnosis – analysis focusing on ALS (including PSMA) patients eTable 8. Long-term exposure to air pollution and risk of being a fast progressor in the overall or domain-specific scores of ALSFRS-R after MND diagnosis – analysis using nonlinear model for fast progressor categorization eTable 9. Long-term exposure to air pollution and risk of being a fast progressor in the overall or domain-specific scores of ALSFRS-R after MND diagnosis – analysis not adjusted for diagnostic delay and ALSFRS-R score at diagnosis [file jamaneurol-e255379-s001.pdf]

# Supplemental Online Content

Wu J, Pyko A, Chourpiliadis C, et al. Long-term exposure to air pollution and risk and prognosis of motor neuron disease. *JAMA Neurol*. Published online January 20, 2026.
doi:10.1001/jamaneurol.2025.5379

**eFigure 1.** Study design
**eFigure 2.** Temporal trend of yearly average concentrations of different air pollutants from 2005 to 2019
**eFigure 3.** The correlation matrix of air pollution at different exposure windows
**eFigure 4.** Observed and predicted individual linear trajectories of ALSFR-S overall and domain-specific scores by time since diagnosis
**eFigure 5.** Observed and predicted individual nonlinear trajectories of ALSFR-S overall and domain-specific scores by time since diagnosis
**eTable 1.** Long-term exposure to air pollution and risk of MND subtypes – analyses of the population comparison
**eTable 2.** Long-term exposure to air pollution and risk of MND – sensitivity analyses in the population comparison
**eTable 3.** Long-term exposure to air pollution in relation to mortality and use of invasive ventilation after MND diagnosis
**eTable 4.** Long-term exposure to air pollution in relation to mortality (or use of invasive ventilation) after MND diagnosis – analysis not adjusted for diagnostic delay and ALSFRS-R score at diagnosis
**eTable 5.** Characteristics of slow and fast progressors in the overall or domain-specific scores of ALSFRS-R
**eTable 6.** Long-term exposure to air pollution in relation to mortality (or use of invasive ventilation) after MND diagnosis – analysis focusing on ALS (including PSMA) patients
**eTable 7.** Long-term exposure to air pollution and risk of being a fast progressor in the overall or domain-specific scores of ALSFRS-R after MND diagnosis – analysis focusing on ALS (including PSMA) patients
**eTable 8.** Long-term exposure to air pollution and risk of being a fast progressor in the overall or domain-specific scores of ALSFRS-R after MND diagnosis – analysis using nonlinear model for fast progressor categorization
**eTable 9.** Long-term exposure to air pollution and risk of being a fast progressor in the overall or domain-specific scores of ALSFRS-R after MND diagnosis – analysis not adjusted for diagnostic delay and ALSFRS-R score at diagnosis

This supplemental material has been provided by the authors to give readers additional information about their work.

© 2026 Wu J et al. JAMA Neurology.

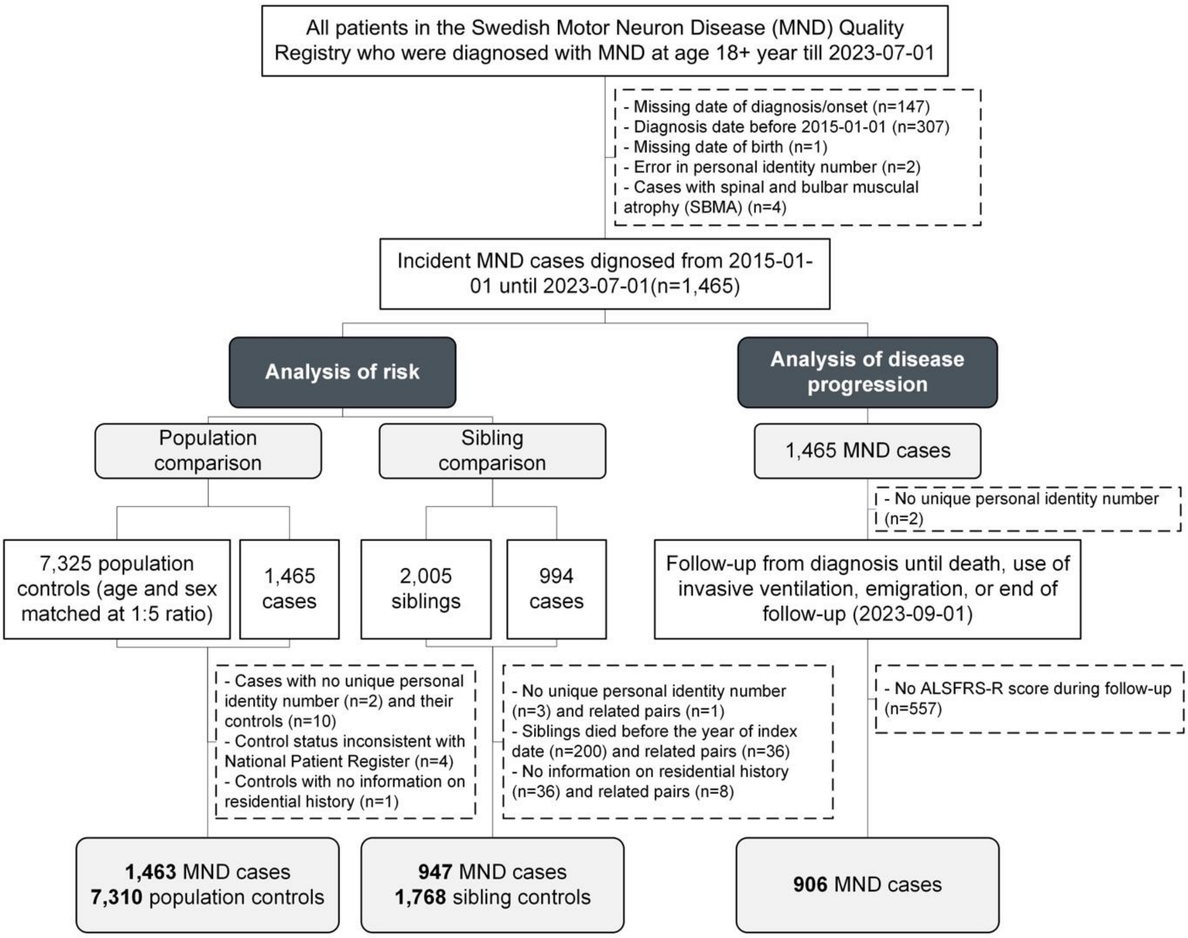


**eFigure 1. Study design**

© 2026 Wu J et al. JAMA Neurology.

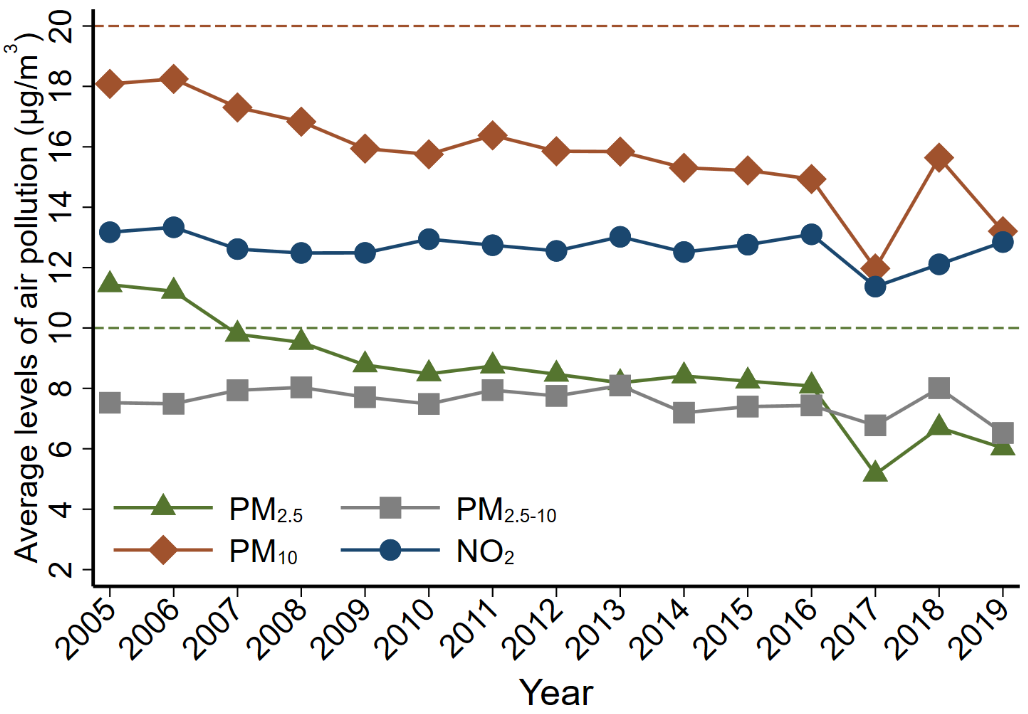


**eFigure 2. Temporal trend of yearly average concentrations of different air pollutants from 2005 to 2019**

*The green and red dashed lines are the 2005 WHO Air Quality Guidelines for $PM_{2.5}$ (10 μg/$m^3$) and $PM_{10}$ (20 μg/$m^3$), respectively. *The 2005 WHO Air Quality Guideline for $NO_2$ was 40 μg/$m^3$.

© 2026 Wu J et al. JAMA Neurology.

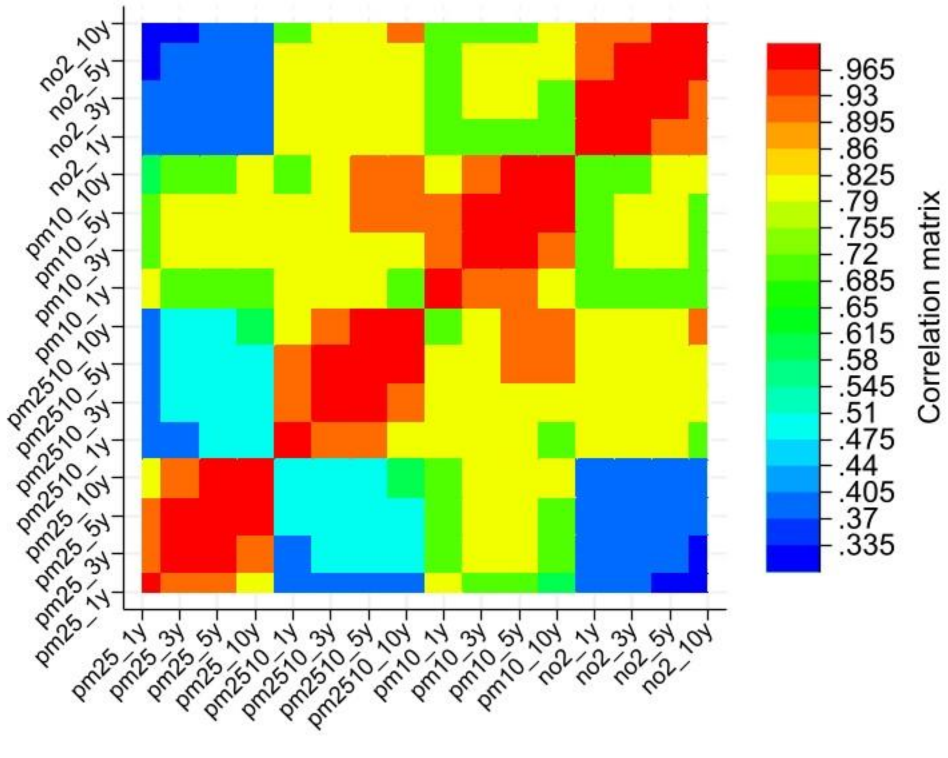


**eFigure 3. The correlation matrix of air pollution at different exposure windows**

© 2026 Wu J et al. JAMA Neurology.

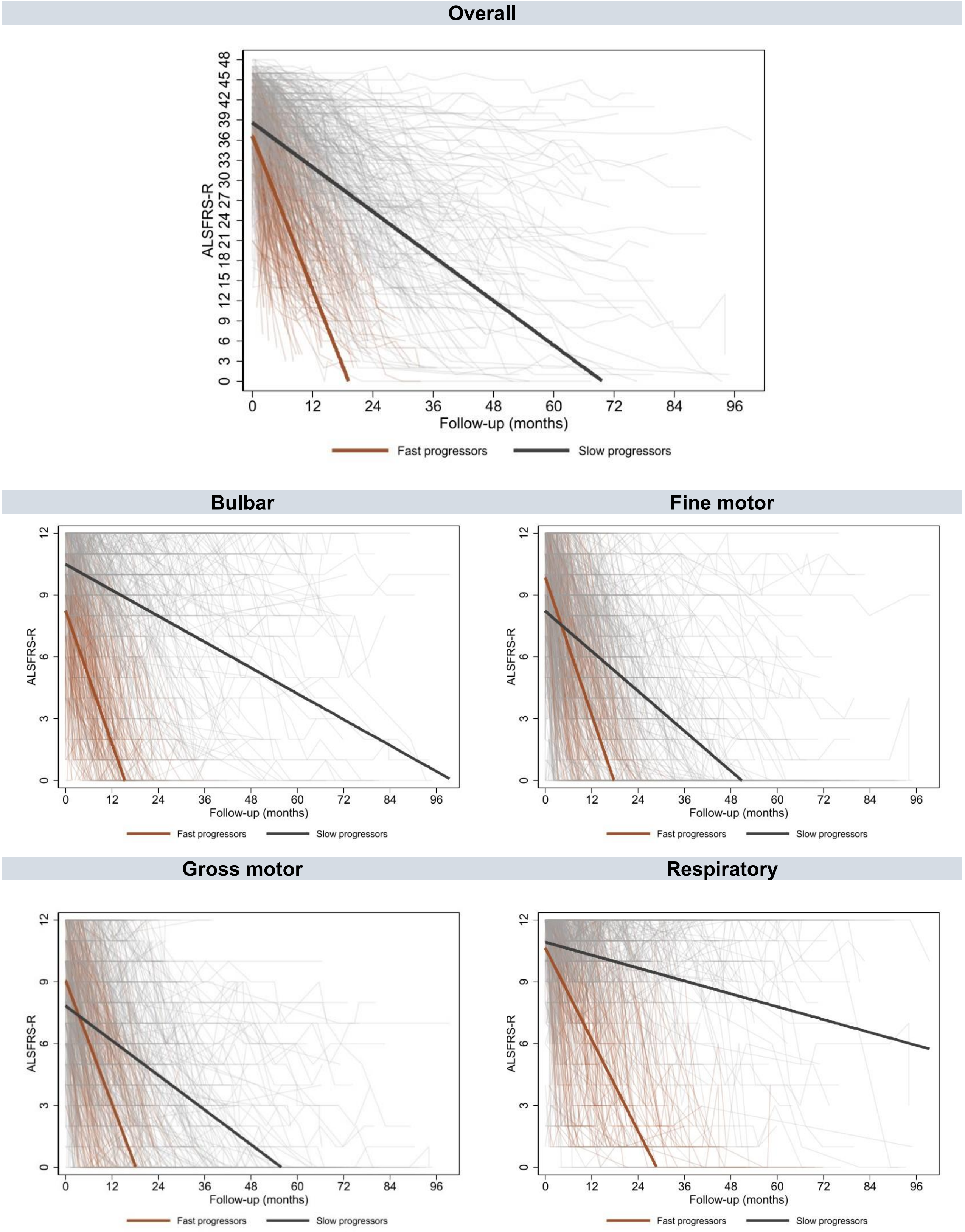


**eFigure 4. Observed and predicted individual *linear* trajectories of ALSFR-S overall and domain-specific scores by time since diagnosis**

© 2026 Wu J et al. JAMA Neurology.

## Overall

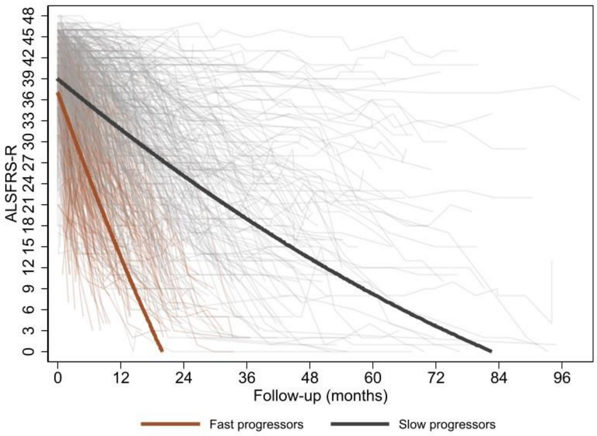


## Bulbar

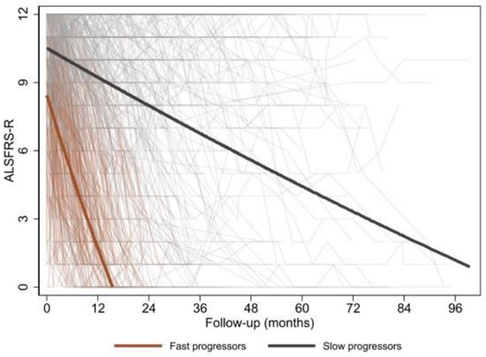


## Fine motor

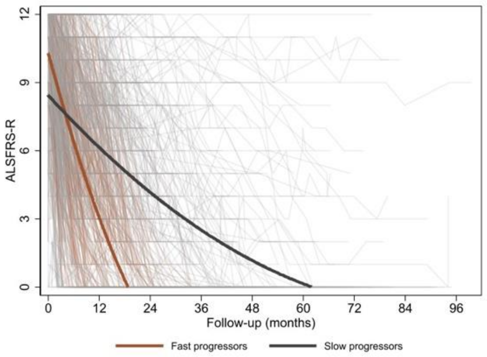


## Gross motor

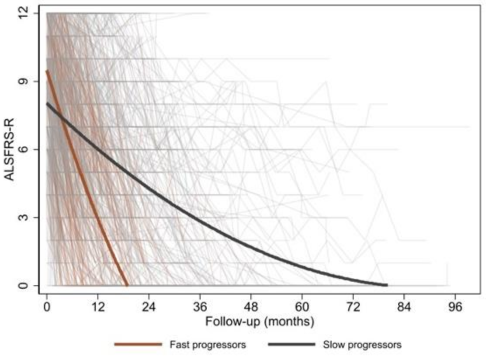


## Respiratory

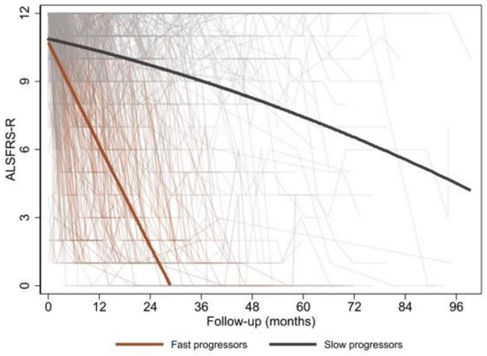


**eFigure 5. Observed and predicted individual *nonlinear* trajectories of ALSFR-S overall and domain-specific scores by time since diagnosis**

© 2026 Wu J et al. JAMA Neurology.

**eTable 1. Long-term exposure to air pollution and risk of MND subtypes – analyses of the population comparison**

| Air pollutants and Exposure windows | ALS (incl. PSMA) | | PLS | | Unspecified MND | |
|---|---|---|---|---|---|---|
| | OR (95%CI) | p-value | OR (95%CI) | p-value | OR (95%CI) | p-value |
| **$PM_{2.5}$ (per IQR: 2 μg/$m^3$)** | | | | | | |
| 1-year average | 1.05 (0.95, 1.18) | 0.337 | 2.18 (0.92, 5.18) | 0.077 | 1.33 (1.10, 1.60) | **0.004** |
| 3-year average | 1.07 (0.95, 1.20) | 0.254 | 1.84 (0.76, 4.48) | 0.179 | 1.34 (1.10, 1.64) | **0.004** |
| 5-year average | 1.09 (0.97, 1.23) | 0.142 | 1.88 (0.78, 4.54) | 0.158 | 1.32 (1.07, 1.61) | **0.008** |
| 10-year average | 1.18 (1.05, 1.33) | **0.006** | 1.92 (0.81, 4.55) | 0.138 | 1.28 (1.04, 1.57) | **0.021** |
| **$PM_{2.5\text{-}10}$ (per IQR: 2.8 μg/$m^3$)** | | | | | | |
| 1-year average | 1.36 (1.23, 1.49) | **0.000** | 1.84 (1.04, 3.26) | **0.036** | 0.88 (0.72, 1.07) | 0.188 |
| 3-year average | 1.37 (1.24, 1.51) | **0.000** | 1.59 (0.89, 2.82) | 0.115 | 0.94 (0.77, 1.14) | 0.515 |
| 5-year average | 1.39 (1.26, 1.53) | **0.000** | 1.66 (0.92, 2.97) | 0.091 | 0.95 (0.77, 1.17) | 0.619 |
| 10-year average | 1.41 (1.27, 1.55) | **0.000** | 1.59 (0.89, 2.83) | 0.119 | 0.93 (0.76, 1.15) | 0.505 |
| **$PM_{10}$ (per IQR: 4 μg/$m^3$)** | | | | | | |
| 1-year average | 1.30 (1.17, 1.44) | **0.000** | 1.76 (0.90, 3.45) | 0.097 | 1.04 (0.86, 1.26) | 0.698 |
| 3-year average | 1.31 (1.18, 1.45) | **0.000** | 1.57 (0.78, 3.13) | 0.203 | 1.09 (0.89, 1.33) | 0.394 |
| 5-year average | 1.31 (1.18, 1.46) | **0.000** | 1.63 (0.82, 3.23) | 0.164 | 1.09 (0.89, 1.34) | 0.392 |
| 10-year average | 1.36 (1.22, 1.51) | **0.000** | 1.62 (0.83, 3.16) | 0.161 | 1.07 (0.88, 1.31) | 0.488 |
| **$NO_2$ (per IQR: 8 μg/$m^3$)** | | | | | | |
| 1-year average | 1.31 (1.21, 1.42) | **0.000** | 1.73 (1.07, 2.82) | **0.026** | 0.84 (0.71, 0.99) | **0.043** |
| 3-year average | 1.32 (1.21, 1.43) | **0.000** | 1.49 (0.90, 2.46) | 0.121 | 0.87 (0.74, 1.03) | 0.109 |
| 5-year average | 1.31 (1.21, 1.42) | **0.000** | 1.42 (0.85, 2.36) | 0.176 | 0.85 (0.72, 1.01) | 0.064 |
| 10-year average | 1.31 (1.21, 1.42) | **0.000** | 1.32 (0.77, 2.26) | 0.307 | 0.84 (0.71, 1.00) | 0.055 |

OR was derived from conditional logistic regression models, after adjustment for age at the index date (matching factor), sex (matching factor), country of birth, education, occupation, household disposable income, and 5-year average neighborhood income before the index date.

Abbreviations: ALS, amyotrophic lateral sclerosis; CI, confidence interval; OR, odds ratio; PM, particulate matter; PLS, primary lateral sclerosis; $NO_2$, nitrogen dioxide; IQR, interquartile range; μg/$m^3$, microgram per cubic meter.

© 2026 Wu J et al. JAMA Neurology.

## eTable 2. Long-term exposure to air pollution and risk of MND – sensitivity analyses in the population comparison

| Air pollutants and Exposure windows | Excluding cases diagnosed between 2021-2023 | | Additional adjustment for urbanicity of the longest living area | |
|---|---|---|---|---|
| | OR (95%CI) | p-value | OR (95%CI) | p-value |
| **$PM_{2.5}$ (per IQR: 2 μg/m$^3$)** | | | | |
| 1-year average | 1.10 (0.98, 1.23) | 0.106 | 1.08 (0.98, 1.20) | 0.116 |
| 3-year average | 1.12 (0.99, 1.26) | 0.084 | 1.09 (0.98, 1.21) | 0.108 |
| 5-year average | 1.14 (1.00, 1.29) | **0.043** | 1.10 (0.99, 1.23) | 0.074 |
| 10-year average | 1.19 (1.05, 1.34) | **0.008** | 1.17 (1.05, 1.30) | **0.006** |
| **$PM_{2.5-10}$ (per IQR: 2.8 μg/m$^3$)** | | | | |
| 1-year average | 1.21 (1.09, 1.35) | **0.000** | 1.22 (1.11, 1.33) | **0.000** |
| 3-year average | 1.25 (1.12, 1.39) | **0.000** | 1.24 (1.13, 1.36) | **0.000** |
| 5-year average | 1.26 (1.13, 1.40) | **0.000** | 1.26 (1.15, 1.39) | **0.000** |
| 10-year average | 1.27 (1.14, 1.41) | **0.000** | 1.28 (1.16, 1.41) | **0.000** |
| **$PM_{10}$ (per IQR: 4 μg/m$^3$)** | | | | |
| 1-year average | 1.19 (1.06, 1.32) | **0.002** | 1.21 (1.10, 1.34) | **0.000** |
| 3-year average | 1.21 (1.08, 1.35) | **0.001** | 1.23 (1.11, 1.36) | **0.000** |
| 5-year average | 1.22 (1.09, 1.37) | **0.001** | 1.24 (1.12, 1.37) | **0.000** |
| 10-year average | 1.25 (1.12, 1.39) | **0.000** | 1.27 (1.15, 1.41) | **0.000** |
| **$NO_2$ (per IQR: 8 μg/m$^3$)** | | | | |
| 1-year average | 1.17 (1.07, 1.27) | **0.000** | 1.18 (1.09, 1.28) | **0.000** |
| 3-year average | 1.19 (1.09, 1.29) | **0.000** | 1.19 (1.10, 1.29) | **0.000** |
| 5-year average | 1.18 (1.08, 1.29) | **0.000** | 1.18 (1.09, 1.28) | **0.000** |
| 10-year average | 1.18 (1.08, 1.29) | **0.000** | 1.18 (1.08, 1.28) | **0.000** |

OR was derived from conditional logistic regression models, after adjustment for age at the index date (matching factor), sex (matching factor), country of birth, education, occupation, household disposable income, and 5-year average neighborhood income before the index date.

Abbreviations: CI, confidence interval; OR, odds ratio; PM, particulate matter; $NO_2$, nitrogen dioxide; IQR, interquartile range; μg/m$^3$, microgram per cubic meter.

© 2026 Wu J et al. JAMA Neurology.

## eTable 3. Long-term exposure to air pollution in relation to mortality and use of invasive ventilation after MND diagnosis

| Air pollutants and Exposure windows | Mortality | | Invasive ventilation | |
|---|---|---|---|---|
| | HR (95%CI) | p-values | HR (95%CI) | p-values |
| $PM_{2.5}$ (per IQR: 2 μg/m$^3$) | | | | |
| 1-year average | 1.03 (0.90, 1.18) | 0.680 | 2.27 (1.00, 5.15) | **0.050** |
| 3-year average | 1.00 (0.86, 1.16) | 0.949 | 2.46 (0.92, 6.55) | 0.071 |
| 5-year average | 1.01 (0.87, 1.18) | 0.896 | 2.55 (0.88, 7.36) | 0.085 |
| 10-year average | 1.00 (0.84, 1.20) | 0.979 | 2.88 (0.94, 8.81) | 0.064 |
| $PM_{2.5-10}$ (per IQR: 2.8 μg/m$^3$) | | | | |
| 1-year average | 1.11 (0.98, 1.26) | 0.089 | 1.85 (0.95, 3.63) | 0.072 |
| 3-year average | 1.10 (0.97, 1.25) | 0.131 | 1.76 (0.88, 3.53) | 0.112 |
| 5-year average | 1.09 (0.96, 1.24) | 0.189 | 1.74 (0.87, 3.48) | 0.119 |
| 10-year average | 1.10 (0.97, 1.25) | 0.151 | 1.52 (0.76, 3.06) | 0.241 |
| $PM_{10}$ (per IQR: 4 μg/m$^3$) | | | | |
| 1-year average | 1.28 (1.11, 1.48) | **0.001** | 1.73 (0.85, 3.52) | 0.128 |
| 3-year average | 1.24 (1.07, 1.45) | **0.005** | 1.78 (0.80, 3.94) | 0.156 |
| 5-year average | 1.19 (1.03, 1.38) | **0.021** | 1.88 (0.83, 4.25) | 0.131 |
| 10-year average | 1.15 (0.99, 1.33) | 0.060 | 1.85 (0.83, 4.12) | 0.131 |
| $NO_2$ (per IQR: 8 μg/m$^3$) | | | | |
| 1-year average | 1.22 (1.10, 1.36) | **0.000** | 1.32 (0.74, 2.36) | 0.350 |
| 3-year average | 1.19 (1.07, 1.32) | **0.001** | 1.26 (0.67, 2.37) | 0.472 |
| 5-year average | 1.15 (1.04, 1.28) | **0.008** | 1.38 (0.72, 2.65) | 0.332 |
| 10-year average | 1.12 (1.01, 1.24) | **0.030** | 1.30 (0.66, 2.56) | 0.446 |

Models were adjusted for age at diagnosis, sex, country of birth, education, occupation, household disposable income, 5-year average neighborhood income, diagnostic delay, and ALSFRS-R score at diagnosis.

Abbreviations: CI, confidence interval; HR, hazard ratio; PM, particulate matter; $NO_2$, nitrogen dioxide; IQR, interquartile range; μg/m$^3$, microgram per cubic meter.

© 2026 Wu J et al. JAMA Neurology.

**eTable 4. Long-term exposure to air pollution in relation to mortality (or use of invasive ventilation) after MND diagnosis – analysis not adjusted for diagnostic delay and ALSFRS-R score at diagnosis**

| Air pollutants and exposure windows | HR (95%CI) | p-value |
|---|---|---|
| $PM_{2.5}$ (per IQR: 2 μg/m$^3$) | | |
| 1-year average | 0.99 (0.90, 1.09) | 0.807 |
| 3-year average | 0.92 (0.83, 1.03) | 0.144 |
| 5-year average | 0.94 (0.84, 1.05) | 0.247 |
| 10-year average | 0.98 (0.87, 1.10) | 0.705 |
| $PM_{2.5-10}$ (per IQR: 2.8 μg/m$^3$) | | |
| 1-year average | 1.16 (1.05, 1.28) | **0.002** |
| 3-year average | 1.18 (1.07, 1.30) | **0.001** |
| 5-year average | 1.19 (1.08, 1.32) | **0.001** |
| 10-year average | 1.21 (1.09, 1.33) | **0.000** |
| $PM_{10}$ (per IQR: 4 μg/m$^3$) | | |
| 1-year average | 1.18 (1.06, 1.30) | **0.002** |
| 3-year average | 1.16 (1.04, 1.29) | **0.007** |
| 5-year average | 1.16 (1.04, 1.29) | **0.009** |
| 10-year average | 1.18 (1.06, 1.31) | **0.003** |
| $NO_2$ (per IQR: 8 μg/m$^3$) | | |
| 1-year average | 1.20 (1.10, 1.30) | **0.000** |
| 3-year average | 1.19 (1.10, 1.29) | **0.000** |
| 5-year average | 1.19 (1.09, 1.29) | **0.000** |
| 10-year average | 1.19 (1.10, 1.29) | **0.000** |

Models were adjusted for age at diagnosis, sex, country of birth, education, occupation, household disposable income, and 5-year average neighborhood income.

Abbreviations: ALSFRS-R, Amyotrophic Lateral Sclerosis Functional Rating Scale-Revised; CI, confidence interval; HR, hazard ratio; PM, particulate matter; $NO_2$, nitrogen dioxide; IQR, interquartile range; μg/m$^3$, microgram per cubic meter.

© 2026 Wu J et al. JAMA Neurology.

## eTable 5. Characteristics of slow and fast progressors in the overall or domain-specific scores of ALSFRS-R

| | Overall score | | | Bulbar | | | Fine motor | | | Gross motor | | | Respiratory | | |
|---|---|---|---|---|---|---|---|---|---|---|---|---|---|---|---|
| | Slow | Fast | p-value | Slow | Fast | p-value | Slow | Fast | p-value | Slow | Fast | p-value | Slow | Fast | p-value |
| Characteristics | N=631 | N=275 | | N=622 | N=284 | | N=682 | N=224 | | N=682 | N=224 | | N=710 | N=196 | |
| **Age at diagnosis** | 65.5±12.6 | 68.2±10.3 | 0.002 | 65.1±12.7 | 69.0±9.6 | <0.001 | 66.0±12.5 | 67.1±10.1 | 0.27 | 66.1±12.6 | 66.7±10.1 | 0.52 | 66.6±12.3 | 65.3±10.9 | 0.20 |
| **Sex** | | | 0.47 | | | 0.006 | | | 0.83 | | | 0.83 | | | 0.11 |
| **Male** | 356 (56.4) | 148 (53.8) | | 365 (58.7) | 139 (48.9) | | 378 (55.4) | 126 (56.2) | | 378 (55.4) | 126 (56.2) | | 385 (54.2) | 119 (60.7) | |
| **Female** | 275 (43.6) | 127 (46.2) | | 257 (41.3) | 145 (51.1) | | 304 (44.6) | 98 (43.8) | | 304 (44.6) | 98 (43.8) | | 325 (45.8) | 77 (39.3) | |
| **Household disposable income** | | | 0.21 | | | 0.38 | | | 0.13 | | | 0.27 | | | 0.14 |
| **Lowest 25%** | 136 (21.6) | 49 (17.8) | | 132 (21.2) | 53 (18.7) | | 150 (22.0) | 35 (15.6) | | 148 (21.7) | 37 (16.5) | | 156 (22.0) | 29 (14.8) | |
| **25-50%** | 122 (19.3) | 56 (20.4) | | 117 (18.8) | 61 (21.5) | | 136 (19.9) | 42 (18.8) | | 137 (20.1) | 41 (18.3) | | 138 (19.4) | 40 (20.4) | |
| **50-75%** | 134 (21.2) | 74 (26.9) | | 136 (21.9) | 72 (25.4) | | 148 (21.7) | 60 (26.8) | | 151 (22.1) | 57 (25.4) | | 156 (22.0) | 52 (26.5) | |
| **Highest 75%** | 239 (37.9) | 96 (34.9) | | 237 (38.1) | 98 (34.5) | | 248 (36.4) | 87 (38.8) | | 246 (36.1) | 89 (39.7) | | 260 (36.6) | 75 (38.3) | |
| **Occupation** | | | 0.29 | | | 0.28 | | | 0.060 | | | 0.045 | | | 0.36 |
| **Occupation without educational requirement** | 21 (3.7) | 8 (3.2) | | 20 (3.6) | 9 (3.4) | | 23 (3.7) | 6 (2.9) | | 23 (3.7) | 6 (2.9) | | 25 (3.9) | 4 (2.2) | |
| **Occupation requiring high school degree** | 261 (45.6) | 106 (42.1) | | 249 (44.2) | 118 (45.2) | | 287 (46.5) | 80 (38.6) | | 289 (47.1) | 78 (37.1) | | 294 (45.6) | 73 (40.8) | |
| **Occupation requiring university studies ≤3 years** | 107 (18.7) | 62 (24.6) | | 107 (19.0) | 62 (23.8) | | 114 (18.5) | 55 (26.6) | | 123 (20.0) | 46 (21.9) | | 131 (20.3) | 38 (21.2) | |

© 2026 Wu J et al. JAMA Neurology.

| | | | | | | | | | | | | | | | |
|---|---|---|---|---|---|---|---|---|---|---|---|---|---|---|---|
| **Occupation requiring university studies >3 years** | 183 (32.0) | 76 (30.2) | | 187 (33.2) | 72 (27.6) | | 193 (31.3) | 66 (31.9) | | 179 (29.2) | 80 (38.1) | | 195 (30.2) | 64 (35.8) | |
| **Education** | | | 0.077 | | | 0.81 | | | 0.43 | | | 0.049 | | | 0.68 |
| **<9 years** | 43 (6.9) | 22 (8.0) | | 44 (7.2) | 21 (7.4) | | 46 (6.8) | 19 (8.5) | | 55 (8.1) | 10 (4.5) | | 50 (7.1) | 15 (7.7) | |
| **9-10 years** | 66 (10.6) | 19 (6.9) | | 58 (9.4) | 27 (9.5) | | 65 (9.6) | 20 (9.0) | | 70 (10.4) | 15 (6.7) | | 69 (9.8) | 16 (8.2) | |
| **Upper secondary education** | 236 (37.8) | 125 (45.6) | | 242 (39.3) | 119 (42.0) | | 268 (39.7) | 93 (41.7) | | 267 (39.6) | 94 (42.2) | | 274 (39.0) | 87 (44.6) | |
| **Post-Secondary <2 years** | 42 (6.7) | 11 (4.0) | | 40 (6.5) | 13 (4.6) | | 46 (6.8) | 7 (3.1) | | 41 (6.1) | 12 (5.4) | | 42 (6.0) | 11 (5.6) | |
| **Post-Secondary ≥2 years** | 229 (36.7) | 91 (33.2) | | 220 (35.8) | 100 (35.3) | | 240 (35.6) | 80 (35.9) | | 235 (34.8) | 85 (38.1) | | 258 (36.7) | 62 (31.8) | |
| **Postgraduate education** | 8 (1.3) | 6 (2.2) | | 11 (1.8) | 3 (1.1) | | 10 (1.5) | 4 (1.8) | | 7 (1.0) | 7 (3.1) | | 10 (1.4) | 4 (2.1) | |
| **Country of birth** | | | 0.40 | | | 0.34 | | | 0.89 | | | 0.45 | | | 0.71 |
| **Sweden** | 542 (85.9) | 228 (82.9) | | 526 (84.6) | 244 (85.9) | | 583 (85.5) | 187 (83.5) | | 574 (84.2) | 196 (87.5) | | 603 (84.9) | 167 (85.2) | |
| **Other Nordic country** | 28 (4.4) | 15 (5.5) | | 26 (4.2) | 17 (6.0) | | 31 (4.5) | 12 (5.4) | | 32 (4.7) | 11 (4.9) | | 33 (4.6) | 10 (5.1) | |
| **Other European country** | 30 (4.8) | 20 (7.3) | | 37 (5.9) | 13 (4.6) | | 36 (5.3) | 14 (6.2) | | 42 (6.2) | 8 (3.6) | | 42 (5.9) | 8 (4.1) | |
| **Non-EU** | 31 (4.9) | 12 (4.4) | | 33 (5.3) | 10 (3.5) | | 32 (4.7) | 11 (4.9) | | 34 (5.0) | 9 (4.0) | | 32 (4.5) | 11 (5.6) | |
| **Mean neighborhood income, SEK thousands** | 355.8 (308.3, 434.7) | 367.9 (316.1, 434.3) | 0.29 | 359.4 (313.5, 425.0) | 367.8 (311.1, 447.9) | 0.26 | 355.7 (309.6, 431.7) | 371.5 (316.0, 445.6) | 0.094 | 355.0 (308.3, 423.3) | 386.8 (322.5, 455.6) | 0.002 | 360.4 (311.9, 428.3) | 368.5 (310.8, 461.3) | 0.17 |
| **BMI at diagnosis, kg/m²** | 24.1±4.1 | 23.9±4.3 | 0.52 | 24.3±4.3 | 23.5±3.9 | 0.015 | 23.9±4.3 | 24.4±3.8 | 0.15 | 23.9±4.2 | 24.3±4.0 | 0.22 | 24.0±4.2 | 24.1±4.1 | 0.75 |

© 2026 Wu J et al. JAMA Neurology.

| | | | | | | | | | | | | | | | |
|---|---|---|---|---|---|---|---|---|---|---|---|---|---|---|---|
| **ALSFRS-R at diagnosis** | 37.2±7.7 | 34.4±9.1 | <0.001 | 37.1±7.9 | 34.7±8.8 | <0.001 | 35.7±8.7 | 38.3±6.5 | <0.001 | 35.9±8.5 | 37.7±7.3 | 0.005 | 36.6±8.1 | 35.5±8.7 | 0.098 |
| **Diagnostic delay (months)** | 19.2±22.4 | 13.2±9.2 | <0.001 | 19.8±22.6 | 12.0±7.7 | <0.001 | 18.5±21.7 | 14.1±10.0 | 0.004 | 18.8±21.7 | 12.9±8.9 | <0.001 | 17.5±18.7 | 16.9±22.3 | 0.73 |
| **Progression rate at diagnosis, median (p25-p75)** | 0.6 (0.3, 1.2) | 0.9 (0.5, 1.8) | <0.001 | 0.6 (0.3, 1.2) | 1.0 (0.6, 1.8) | <0.001 | 0.8 (0.3, 1.5) | 0.7 (0.4, 1.1) | 0.31 | 0.7 (0.3, 1.4) | 0.8 (0.4, 1.4) | 0.20 | 0.7 (0.3, 1.4) | 0.8 (0.4, 1.5) | 0.14 |
| **Family history** | 36 (10.3) | 16 (10.7) | 0.89 | 35 (10.1) | 17 (11.3) | 0.67 | 38 (10.8) | 14 (9.5) | 0.66 | 39 (10.9) | 13 (9.4) | 0.62 | 38 (9.9) | 14 (12.1) | 0.51 |
| **Onset site** | | | 0.10 | | | <0.001 | | | 0.16 | | | 0.57 | | | 0.014 |
| **Bulbar** | 145 (31.1) | 85 (39.4) | | 114 (24.5) | 116 (53.5) | | 156 (31.8) | 74 (38.5) | | 162 (32.7) | 68 (36.6) | | 193 (36.6) | 37 (24.0) | |
| **Spinal** | 287 (61.6) | 118 (54.6) | | 317 (68.2) | 88 (40.6) | | 302 (61.6) | 103 (53.6) | | 298 (60.1) | 107 (57.5) | | 299 (56.6) | 106 (68.8) | |
| **Other** | 34 (7.3) | 13 (6.0) | | 34 (7.3) | 13 (6.0) | | 32 (6.5) | 15 (7.8) | | 36 (7.3) | 11 (5.9) | | 36 (6.8) | 11 (7.1) | |
| **Invasive ventilation** | 18 (2.9) | 1 (0.4) | 0.016 | 18 (2.9) | 1 (0.4) | 0.013 | 18 (2.6) | 1 (0.4) | 0.047 | 18 (2.6) | 1 (0.4) | 0.047 | 11 (1.5) | 8 (4.1) | 0.029 |
| **Time from diagnosis to death in months** | 23.2±18.0 | 13.2±8.5 | <0.001 | 22.9±18.2 | 13.7±8.4 | <0.001 | 20.3±17.8 | 16.6±9.2 | 0.006 | 20.2±17.7 | 16.8±9.6 | 0.012 | 18.9±16.5 | 19.7±13.0 | 0.54 |
| **Gastrostomy** | | | 0.81 | | | 0.51 | | | 0.072 | | | 0.41 | | | 0.50 |
| **PEG** | 110 (97.3) | 92 (97.9) | | 91 (96.8) | 111 (98.2) | | 122 (96.1) | 80 (100.0) | | 125 (96.9) | 77 (98.7) | | 132 (97.1) | 70 (98.6) | |
| **RIG** | 3 (2.7) | 2 (2.1) | | 3 (3.2) | 2 (1.8) | | 5 (3.9) | 0 (0.0) | | 4 (3.1) | 1 (1.3) | | 4 (2.9) | 1 (1.4) | |
| **1-year average $PM_{2.5}$** | 6.7±1.5 | 6.7±1.4 | 0.48 | 6.7±1.4 | 6.7±1.4 | 0.61 | 6.8±1.4 | 6.6±1.4 | 0.23 | 6.8±1.5 | 6.6±1.4 | 0.076 | 6.7±1.4 | 6.8±1.5 | 0.56 |
| **1-year average $PM_{10}$** | 14.7±2.9 | 14.8±2.7 | 0.74 | 14.8±2.8 | 14.8±2.9 | 0.98 | 14.7±2.8 | 15.0±3.0 | 0.21 | 14.8±2.8 | 14.7±2.9 | 0.88 | 14.8±2.8 | 14.8±2.8 | 0.76 |
| **1-year average $PM_{2.5-10}$** | 7.6±2.3 | 7.8±2.3 | 0.21 | 7.7±2.3 | 7.7±2.4 | 0.89 | 7.5±2.2 | 8.0±2.4 | 0.006 | 7.6±2.3 | 7.9±2.4 | 0.074 | 7.6±2.3 | 7.9±2.3 | 0.14 |
| **1-year average $NO_2$** | 14.7±7.5 | 15.7±7.6 | 0.060 | 14.7±7.4 | 15.5±7.7 | 0.12 | 14.6±7.3 | 16.2±8.1 | 0.004 | 14.7±7.4 | 15.7±7.9 | 0.12 | 14.9±7.5 | 15.1±7.7 | 0.75 |
| **3-year average $PM_{2.5}$** | 7.0±1.4 | 7.1±1.3 | 0.40 | 7.0±1.3 | 7.0±1.3 | 0.58 | 7.0±1.3 | 7.0±1.3 | 0.68 | 7.0±1.4 | 7.0±1.3 | 0.71 | 7.0±1.3 | 7.1±1.3 | 0.094 |

© 2026 Wu J et al. JAMA Neurology.

| | | | | | | | | | | | | | | | |
|---|---|---|---|---|---|---|---|---|---|---|---|---|---|---|---|
| **3-year average $PM_{10}$** | 15.0±2.7 | 15.3±2.6 | 0.17 | 15.0±2.6 | 15.1±2.9 | 0.79 | 15.0±2.6 | 15.3±2.8 | 0.094 | 15.0±2.7 | 15.2±2.8 | 0.42 | 15.0±2.7 | 15.1±2.7 | 0.66 |
| **3-year average $PM_{2.5-10}$** | 7.7±2.2 | 8.1±2.3 | 0.040 | 7.8±2.2 | 7.9±2.4 | 0.51 | 7.7±2.2 | 8.2±2.4 | 0.005 | 7.7±2.2 | 8.2±2.3 | 0.013 | 7.8±2.2 | 8.1±2.3 | 0.12 |
| **3-year average $NO_2$** | 14.6±7.4 | 15.8±7.7 | 0.028 | 14.6±7.3 | 15.7±7.9 | 0.058 | 14.6±7.3 | 16.2±8.0 | 0.005 | 14.7±7.3 | 15.8±8.0 | 0.046 | 14.9±7.4 | 15.1±7.9 | 0.76 |
| **5-year average $PM_{2.5}$** | 7.3±1.3 | 7.4±1.2 | 0.15 | 7.3±1.3 | 7.3±1.3 | 0.88 | 7.3±1.3 | 7.4±1.3 | 0.30 | 7.3±1.3 | 7.3±1.2 | 0.82 | 7.3±1.3 | 7.5±1.3 | 0.029 |
| **5-year average $PM_{10}$** | 15.3±2.7 | 15.6±2.6 | 0.11 | 15.3±2.6 | 15.4±2.8 | 0.72 | 15.3±2.6 | 15.6±2.8 | 0.093 | 15.3±2.7 | 15.5±2.7 | 0.44 | 15.3±2.7 | 15.4±2.7 | 0.64 |
| **5-year average $PM_{2.5-10}$** | 7.9±2.1 | 8.2±2.3 | 0.021 | 8.0±2.1 | 8.1±2.3 | 0.41 | 7.9±2.1 | 8.3±2.3 | 0.007 | 7.9±2.1 | 8.3±2.3 | 0.022 | 7.9±2.1 | 8.2±2.3 | 0.23 |
| **5-year average $NO_2$** | 14.6±7.4 | 15.7±7.7 | 0.033 | 14.6±7.3 | 15.6±7.8 | 0.069 | 14.5±7.3 | 16.1±8.0 | 0.005 | 14.6±7.3 | 15.8±7.9 | 0.044 | 14.8±7.3 | 15.1±8.0 | 0.65 |
| **10-year average $PM_{2.5}$** | 8.1±1.2 | 8.3±1.0 | 0.039 | 8.1±1.2 | 8.2±1.1 | 0.38 | 8.1±1.2 | 8.3±1.1 | 0.18 | 8.1±1.2 | 8.2±1.0 | 0.39 | 8.1±1.2 | 8.3±1.1 | 0.013 |
| **10-year average $PM_{10}$** | 16.1±2.8 | 16.5±2.6 | 0.031 | 16.2±2.7 | 16.4±2.8 | 0.33 | 16.1±2.7 | 16.5±2.8 | 0.073 | 16.2±2.8 | 16.4±2.7 | 0.23 | 16.2±2.7 | 16.3±2.8 | 0.49 |
| **10-year average $PM_{2.5-10}$** | 8.1±2.2 | 8.6±2.3 | 0.010 | 8.2±2.2 | 8.4±2.3 | 0.26 | 8.2±2.2 | 8.6±2.4 | 0.016 | 8.2±2.2 | 8.5±2.3 | 0.033 | 8.2±2.2 | 8.4±2.4 | 0.39 |
| **10-year average $NO_2$** | 14.7±7.4 | 15.8±7.7 | 0.041 | 14.7±7.3 | 15.7±7.9 | 0.079 | 14.7±7.3 | 16.1±8.0 | 0.017 | 14.8±7.4 | 15.8±7.9 | 0.080 | 15.0±7.3 | 15.2±8.1 | 0.66 |

Data are presented as mean±SD or median (IQR) for continuous measures, and n (%) for categorical measures.

[#]Mean neighborhood income was calculated as 5-year average before the date of diagnosis.

Abbreviations: ALSFRS-R, Amyotrophic Lateral Sclerosis Functional Rating Scale-Revised; BMI, body mass index; PM, particulate matter; PEG, percutaneous endoscopic gastrostomy; $NO_2$, nitrogen dioxide; IQR, interquartile range; $\mu g/m^3$, microgram per cubic meter; RIG, radiologically inserted gastrostomy

© 2026 Wu J et al. JAMA Neurology.

**eTable 6. Long-term exposure to air pollution in relation to mortality (or use of invasive ventilation) after MND diagnosis – analysis focusing on ALS (including PSMA) patients**

| **Air pollutants and Exposure windows** | HR (95%CI) | p-value |
|---|---|---|
| **$PM_{2.5}$ (per IQR: 2 μg/m³)** | | |
| 1-year average | 1.10 (0.94, 1.30) | 0.245 |
| 3-year average | 1.03 (0.86, 1.23) | 0.777 |
| 5-year average | 1.04 (0.86, 1.26) | 0.669 |
| 10-year average | 1.02 (0.82, 1.26) | 0.856 |
| **$PM_{2.5-10}$ (per IQR: 2.8 μg/m³)** | | |
| 1-year average | 1.13 (0.99, 1.29) | 0.076 |
| 3-year average | 1.11 (0.97, 1.27) | 0.138 |
| 5-year average | 1.10 (0.96, 1.27) | 0.173 |
| 10-year average | 1.09 (0.95, 1.25) | 0.226 |
| **$PM_{10}$ (per IQR: 4 μg/m³)** | | |
| 1-year average | 1.35 (1.16, 1.58) | **0.000** |
| 3-year average | 1.28 (1.09, 1.51) | **0.003** |
| 5-year average | 1.22 (1.04, 1.44) | **0.015** |
| 10-year average | 1.15 (0.98, 1.35) | 0.078 |
| **$NO_2$ (per IQR: 8 μg/m³)** | | |
| 1-year average | 1.20 (1.07, 1.35) | **0.002** |
| 3-year average | 1.17 (1.04, 1.32) | **0.007** |
| 5-year average | 1.14 (1.02, 1.28) | **0.025** |
| 10-year average | 1.10 (0.99, 1.24) | 0.088 |

Analyses were adjusted for age at diagnosis, sex, country of birth, education, occupation, household disposable income, 5-year average neighborhood income, diagnostic delay, and ALSFRS-R score at diagnosis.

Abbreviations: CI, confidence interval; HR, hazard ratio; PM, particulate matter; $NO_2$, nitrogen dioxide; IQR, interquartile range; μg/m³, microgram per cubic meter.

© 2026 Wu J et al. JAMA Neurology.

**eTable 7. Long-term exposure to air pollution and risk of being a fast progressor in the overall or domain-specific scores of ALSFRS-R after MND diagnosis – analysis focusing on ALS (including PSMA) patients**

| | Overall | | Bulbar | | Fine motor | | Gross motor | | Respiratory | |
|---|---|---|---|---|---|---|---|---|---|---|
| Air pollutants and Exposure windows | OR (95%CI) | p-value | OR (95%CI) | p-value | OR (95%CI) | p-value | OR (95%CI) | p-value | OR (95%CI) | p-value |
| **$PM_{2.5}$ (per IQR: 2 $\mu g/m^3$)** | | | | | | | | | | |
| 1-year average | 0.88 (0.68, 1.14) | 0.336 | 1.07 (0.83, 1.38) | 0.609 | 1.10 (0.84, 1.45) | 0.487 | 0.82 (0.62, 1.08) | 0.153 | 0.90 (0.67, 1.19) | 0.456 |
| 3-year average | 1.05 (0.79, 1.39) | 0.749 | 1.04 (0.78, 1.37) | 0.795 | 1.31 (0.97, 1.76) | 0.077 | 0.92 (0.68, 1.23) | 0.570 | 1.01 (0.74, 1.37) | 0.965 |
| 5-year average | 1.15 (0.86, 1.54) | 0.352 | 1.11 (0.83, 1.49) | 0.466 | 1.32 (0.97, 1.80) | 0.075 | 0.98 (0.72, 1.32) | 0.883 | 1.05 (0.76, 1.44) | 0.776 |
| 10-year average | 1.30 (0.93, 1.82) | 0.125 | 1.19 (0.85, 1.66) | 0.307 | 1.47 (1.03, 2.08) | **0.033** | 1.08 (0.76, 1.53) | 0.662 | 1.11 (0.77, 1.59) | 0.589 |
| **$PM_{2.5\text{-}10}$ (per IQR: 2.8 $\mu g/m^3$)** | | | | | | | | | | |
| 1-year average | 1.13 (0.91, 1.40) | 0.285 | 1.06 (0.85, 1.31) | 0.613 | 1.47 (1.17, 1.84) | **0.001** | 1.06 (0.85, 1.32) | 0.610 | 1.20 (0.95, 1.52) | 0.124 |
| 3-year average | 1.18 (0.94, 1.48) | 0.145 | 1.11 (0.89, 1.39) | 0.345 | 1.46 (1.16, 1.85) | **0.001** | 1.13 (0.90, 1.42) | 0.275 | 1.17 (0.92, 1.49) | 0.188 |
| 5-year average | 1.21 (0.97, 1.52) | 0.097 | 1.09 (0.87, 1.37) | 0.455 | 1.44 (1.14, 1.83) | **0.003** | 1.09 (0.87, 1.38) | 0.451 | 1.11 (0.87, 1.41) | 0.408 |
| 10-year average | 1.29 (1.03, 1.61) | **0.029** | 1.07 (0.86, 1.34) | 0.545 | 1.34 (1.06, 1.70) | **0.013** | 1.07 (0.85, 1.34) | 0.587 | 1.06 (0.83, 1.35) | 0.652 |
| **$PM_{10}$ (per IQR: 4 $\mu g/m^3$)** | | | | | | | | | | |
| 1-year average | 1.04 (0.81, 1.33) | 0.783 | 1.09 (0.85, 1.39) | 0.509 | 1.30 (1.00, 1.70) | 0.051 | 0.89 (0.68, 1.15) | 0.367 | 0.99 (0.75, 1.31) | 0.959 |
| 3-year average | 1.13 (0.87, 1.48) | 0.351 | 1.12 (0.86, 1.45) | 0.404 | 1.41 (1.06, 1.87) | **0.019** | 0.98 (0.75, 1.29) | 0.898 | 1.00 (0.75, 1.35) | 0.975 |
| 5-year average | 1.16 (0.89, 1.51) | 0.286 | 1.08 (0.83, 1.40) | 0.568 | 1.38 (1.04, 1.83) | **0.027** | 0.96 (0.73, 1.26) | 0.749 | 0.98 (0.73, 1.31) | 0.867 |
| 10-year average | 1.27 (0.98, 1.65) | 0.071 | 1.08 (0.84, 1.40) | 0.542 | 1.35 (1.02, 1.78) | **0.033** | 1.00 (0.76, 1.31) | 0.991 | 1.00 (0.75, 1.33) | 1.000 |
| **$NO_2$ (per IQR: 8 $\mu g/m^3$)** | | | | | | | | | | |
| 1-year average | 1.14 (0.94, 1.38) | 0.170 | 1.13 (0.93, 1.36) | 0.207 | 1.37 (1.12, 1.68) | **0.002** | 1.03 (0.85, 1.26) | 0.749 | 1.09 (0.88, 1.35) | 0.418 |
| 3-year average | 1.13 (0.94, 1.37) | 0.196 | 1.15 (0.96, 1.39) | 0.134 | 1.34 (1.09, 1.64) | **0.005** | 1.07 (0.88, 1.30) | 0.479 | 1.05 (0.85, 1.30) | 0.632 |
| 5-year average | 1.12 (0.92, 1.35) | 0.252 | 1.12 (0.93, 1.36) | 0.222 | 1.32 (1.08, 1.61) | **0.007** | 1.05 (0.87, 1.28) | 0.603 | 1.05 (0.85, 1.29) | 0.673 |
| 10-year average | 1.13 (0.94, 1.37) | 0.198 | 1.09 (0.91, 1.32) | 0.354 | 1.28 (1.05, 1.56) | **0.016** | 1.02 (0.84, 1.24) | 0.833 | 1.03 (0.83, 1.27) | 0.798 |

Models were adjusted for age at diagnosis, sex, education, occupation, household disposable income, 5-year average neighborhood income, diagnostic delay, and ALSFRS-R score at diagnosis.

Abbreviations: ALSFRS-R, Amyotrophic Lateral Sclerosis Functional Rating Scale-Revised; CI, confidence interval; OR, odds ratio; PM, particulate matter; $NO_2$, nitrogen dioxide; IQR, interquartile range; $\mu g/m^3$, microgram per cubic meter.

© 2026 Wu J et al. JAMA Neurology.

**eTable 8. Long-term exposure to air pollution and risk of being a fast progressor in the overall or domain-specific scores of ALSFRS-R after MND diagnosis – analysis using nonlinear model for fast progressor categorization**

| Air pollutants and Exposure windows | Overall | | Bulbar | | Fine motor | | Gross motor | | Respiratory | |
|---|---|---|---|---|---|---|---|---|---|---|
| | OR (95%CI) | p-value | OR (95%CI) | p-value | OR (95%CI) | p-value | OR (95%CI) | p-value | OR (95%CI) | p-value |
| **$PM_{2.5}$ (per IQR: 2 $\mu g/m^3$)** | | | | | | | | | | |
| 1-year average | 0.96 (0.77, 1.20) | 0.722 | 1.01 (0.80, 1.26) | 0.963 | 1.09 (0.85, 1.40) | 0.485 | 0.96 (0.75, 1.22) | 0.729 | 1.12 (0.88, 1.42) | 0.369 |
| 3-year average | 1.14 (0.90, 1.45) | 0.275 | 0.96 (0.75, 1.22) | 0.725 | 1.26 (0.97, 1.63) | 0.086 | 1.07 (0.83, 1.38) | 0.594 | 1.29 (0.99, 1.66) | 0.056 |
| 5-year average | 1.23 (0.96, 1.58) | 0.094 | 1.01 (0.79, 1.29) | 0.915 | 1.32 (1.01, 1.73) | **0.039** | 1.16 (0.90, 1.50) | 0.259 | 1.35 (1.04, 1.76) | **0.026** |
| 10-year average | 1.36 (1.03, 1.80) | **0.031** | 1.08 (0.82, 1.43) | 0.589 | 1.42 (1.05, 1.93) | **0.023** | 1.25 (0.93, 1.68) | 0.137 | 1.46 (1.08, 1.98) | **0.014** |
| **$PM_{2.5\text{-}10}$ (per IQR: 2.8 $\mu g/m^3$)** | | | | | | | | | | |
| 1-year average | 1.17 (0.97, 1.43) | 0.108 | 1.04 (0.86, 1.27) | 0.674 | 1.53 (1.24, 1.89) | **0.000** | 1.23 (1.01, 1.51) | **0.044** | 1.17 (0.95, 1.44) | 0.140 |
| 3-year average | 1.26 (1.02, 1.54) | **0.028** | 1.11 (0.91, 1.36) | 0.297 | 1.62 (1.30, 2.01) | **0.000** | 1.33 (1.08, 1.64) | **0.008** | 1.18 (0.95, 1.46) | 0.135 |
| 5-year average | 1.30 (1.05, 1.60) | **0.014** | 1.12 (0.91, 1.38) | 0.276 | 1.59 (1.27, 1.99) | **0.000** | 1.30 (1.05, 1.61) | **0.018** | 1.13 (0.90, 1.40) | 0.297 |
| 10-year average | 1.32 (1.08, 1.63) | **0.008** | 1.13 (0.92, 1.39) | 0.256 | 1.49 (1.19, 1.85) | **0.000** | 1.24 (1.00, 1.54) | **0.046** | 1.06 (0.85, 1.33) | 0.580 |
| **$PM_{10}$ (per IQR: 4 $\mu g/m^3$)** | | | | | | | | | | |
| 1-year average | 1.07 (0.85, 1.34) | 0.559 | 1.06 (0.85, 1.33) | 0.592 | 1.41 (1.10, 1.80) | **0.007** | 1.08 (0.85, 1.38) | 0.512 | 1.05 (0.82, 1.34) | 0.702 |
| 3-year average | 1.21 (0.95, 1.54) | 0.129 | 1.07 (0.85, 1.36) | 0.560 | 1.55 (1.18, 2.02) | **0.001** | 1.18 (0.92, 1.53) | 0.194 | 1.07 (0.82, 1.39) | 0.605 |
| 5-year average | 1.24 (0.97, 1.57) | 0.087 | 1.05 (0.83, 1.33) | 0.686 | 1.50 (1.15, 1.96) | **0.003** | 1.17 (0.91, 1.51) | 0.220 | 1.06 (0.81, 1.37) | 0.678 |
| 10-year average | 1.32 (1.04, 1.68) | **0.023** | 1.09 (0.86, 1.39) | 0.460 | 1.46 (1.13, 1.90) | **0.004** | 1.18 (0.92, 1.52) | 0.192 | 1.07 (0.82, 1.38) | 0.630 |
| **$NO_2$ (per IQR: 8 $\mu g/m^3$)** | | | | | | | | | | |
| 1-year average | 1.16 (0.98, 1.38) | 0.086 | 1.14 (0.96, 1.36) | 0.127 | 1.46 (1.21, 1.76) | **0.000** | 1.14 (0.96, 1.37) | 0.143 | 1.03 (0.86, 1.25) | 0.733 |
| 3-year average | 1.15 (0.97, 1.37) | 0.110 | 1.15 (0.97, 1.37) | 0.115 | 1.41 (1.17, 1.70) | **0.000** | 1.17 (0.97, 1.40) | 0.097 | 1.01 (0.83, 1.21) | 0.957 |
| 5-year average | 1.14 (0.96, 1.36) | 0.136 | 1.13 (0.95, 1.34) | 0.172 | 1.40 (1.16, 1.69) | **0.000** | 1.16 (0.97, 1.39) | 0.099 | 1.00 (0.83, 1.21) | 0.970 |
| 10-year average | 1.13 (0.95, 1.35) | 0.166 | 1.12 (0.94, 1.33) | 0.205 | 1.34 (1.11, 1.61) | **0.002** | 1.12 (0.94, 1.34) | 0.216 | 0.98 (0.81, 1.19) | 0.856 |

Models were adjusted for age at diagnosis, sex, education, occupation, household disposable income, 5-year average neighborhood income, diagnostic delay, and ALSFRS-R score at diagnosis.

Abbreviations: ALSFRS-R, Amyotrophic Lateral Sclerosis Functional Rating Scale-Revised; CI, confidence interval; OR, odds ratio; PM, particulate matter; $NO_2$, nitrogen dioxide; IQR, interquartile range; $\mu g/m^3$, microgram per cubic meter.

© 2026 Wu J et al. JAMA Neurology.

**eTable 9. Long-term exposure to air pollution and risk of being a fast progressor in the overall or domain-specific scores of ALSFRS-R after MND diagnosis – analysis not adjusted for diagnostic delay and ALSFRS-R score at diagnosis**

| Air pollutants and exposure windows | Overall | | Bulbar | | Fine motor | | Gross motor | | Respiratory | |
|---|---|---|---|---|---|---|---|---|---|---|
| | OR (95%CI) | p-value | OR (95%CI) | p-value | OR (95%CI) | p-value | OR (95%CI) | p-value | OR (95%CI) | p-value |
| **$PM_{2.5}$ (per IQR: 2 μg/$m^3$)** | | | | | | | | | | |
| 1-year average | 1.01 (0.81, 1.25) | 0.956 | 1.03 (0.83, 1.27) | 0.812 | 0.98 (0.78, 1.23) | 0.846 | 0.86 (0.69, 1.09) | 0.215 | 1.15 (0.90, 1.46) | 0.264 |
| 3-year average | 1.19 (0.95, 1.50) | 0.134 | 0.99 (0.79, 1.24) | 0.938 | 1.13 (0.89, 1.45) | 0.322 | 0.98 (0.77, 1.25) | 0.866 | 1.31 (1.01, 1.69) | **0.041** |
| 5-year average | 1.28 (1.01, 1.62) | **0.042** | 1.05 (0.84, 1.33) | 0.659 | 1.19 (0.92, 1.53) | 0.178 | 1.04 (0.81, 1.34) | 0.741 | 1.37 (1.05, 1.78) | **0.020** |
| 10-year average | 1.42 (1.09, 1.86) | **0.010** | 1.13 (0.86, 1.46) | 0.379 | 1.23 (0.93, 1.64) | 0.152 | 1.12 (0.84, 1.48) | 0.440 | 1.49 (1.10, 2.00) | **0.009** |
| **$PM_{2.5\text{-}10}$ (per IQR: 2.8 μg/$m^3$)** | | | | | | | | | | |
| 1-year average | 1.19 (0.98, 1.43) | 0.077 | 1.05 (0.87, 1.27) | 0.582 | 1.41 (1.15, 1.72) | **0.001** | 1.22 (1.00, 1.48) | **0.050** | 1.18 (0.96, 1.45) | 0.122 |
| 3-year average | 1.29 (1.06, 1.57) | **0.011** | 1.14 (0.94, 1.39) | 0.174 | 1.44 (1.17, 1.77) | **0.001** | 1.32 (1.07, 1.61) | **0.008** | 1.20 (0.97, 1.49) | 0.091 |
| 5-year average | 1.32 (1.08, 1.62) | **0.006** | 1.15 (0.94, 1.40) | 0.170 | 1.41 (1.14, 1.74) | **0.001** | 1.27 (1.04, 1.57) | **0.022** | 1.15 (0.92, 1.44) | 0.207 |
| 10-year average | 1.35 (1.11, 1.65) | **0.003** | 1.16 (0.95, 1.41) | 0.142 | 1.34 (1.09, 1.65) | **0.006** | 1.23 (1.00, 1.51) | **0.048** | 1.09 (0.88, 1.36) | 0.425 |
| **$PM_{10}$ (per IQR: 4 μg/$m^3$)** | | | | | | | | | | |
| 1-year average | 1.11 (0.89, 1.39) | 0.342 | 1.08 (0.87, 1.35) | 0.466 | 1.28 (1.01, 1.62) | **0.042** | 1.03 (0.81, 1.29) | 0.830 | 1.07 (0.83, 1.36) | 0.612 |
| 3-year average | 1.28 (1.01, 1.62) | **0.042** | 1.11 (0.88, 1.40) | 0.369 | 1.36 (1.06, 1.76) | **0.016** | 1.15 (0.90, 1.47) | 0.277 | 1.10 (0.84, 1.42) | 0.489 |
| 5-year average | 1.30 (1.03, 1.64) | **0.030** | 1.09 (0.87, 1.37) | 0.453 | 1.33 (1.03, 1.71) | **0.026** | 1.12 (0.88, 1.43) | 0.366 | 1.08 (0.84, 1.41) | 0.541 |
| 10-year average | 1.38 (1.10, 1.74) | **0.006** | 1.14 (0.91, 1.43) | 0.251 | 1.29 (1.01, 1.65) | **0.039** | 1.14 (0.90, 1.45) | 0.281 | 1.10 (0.85, 1.42) | 0.458 |
| **$NO_2$ (per IQR: 8 μg/$m^3$)** | | | | | | | | | | |
| 1-year average | 1.21 (1.02, 1.43) | **0.028** | 1.16 (0.99, 1.37) | 0.074 | 1.38 (1.15, 1.65) | **0.000** | 1.15 (0.97, 1.38) | 0.110 | 1.04 (0.86, 1.26) | 0.670 |
| 3-year average | 1.21 (1.02, 1.43) | **0.025** | 1.18 (1.00, 1.40) | **0.046** | 1.34 (1.12, 1.60) | **0.001** | 1.18 (0.99, 1.41) | 0.059 | 1.02 (0.84, 1.23) | 0.850 |
| 5-year average | 1.20 (1.02, 1.42) | **0.030** | 1.17 (0.99, 1.38) | 0.063 | 1.32 (1.11, 1.58) | **0.002** | 1.18 (0.99, 1.40) | 0.070 | 1.02 (0.85, 1.23) | 0.808 |
| 10-year average | 1.20 (1.01, 1.42) | **0.038** | 1.16 (0.98, 1.37) | 0.079 | 1.26 (1.05, 1.50) | **0.011** | 1.14 (0.95, 1.36) | 0.149 | 1.01 (0.84, 1.22) | 0.913 |

Models were adjusted for age at diagnosis, sex, education, occupation, household disposable income, and 5-year average neighborhood income.

Abbreviations: ALSFRS-R, Amyotrophic Lateral Sclerosis Functional Rating Scale-Revised; CI, confidence interval; OR, odds ratio; PM, particulate matter; $NO_2$, nitrogen dioxide; IQR, interquartile range; μg/$m^3$, microgram per cubic meter.

© 2026 Wu J et al. JAMA Neurology.
