# Supplementary material for: Long-Term Exposure to Air Pollution and Risk and Prognosis of Motor Neuron Disease
Source: JAMA Neurol. 2026 Jan 20;83(3):213–22. doi: 10.1001/jamaneurol.2025.5379 (PMC12820776; doi:10.1001/jamaneurol.2025.5379)
Supplement: Supplement 2. — Data Sharing Statement [file jamaneurol-e255379-s002.pdf]

Data Sharing Statement

Wu. Long-Term Exposure to Air Pollution and Risk and Prognosis of Motor Neuron Disease. *JAMA Neurol*. Published January 20, 2026. doi:10.1001/jamaneurol.2025.5379

Data
Data available: No

Additional Information
Explanation for why data not available: The data are not publicly available due to Swedish and European regulations. The aggregated data that support the findings of this study are available from the corresponding author upon reasonable request.
